# The effect of COVID-19 risk perception on pro-environmental behavior of Chinese consumers: Perspectives from affective event theory

**DOI:** 10.3389/fpsyg.2022.1093999

**Published:** 2023-01-04

**Authors:** Shuai Zhou

**Affiliations:** ^1^School of Economics and Management, Zhoukou Normal University, Zhoukou, Henan, China; ^2^Asia-Europe Institute, University of Malaya, Kuala Lumpur, Malaysia

**Keywords:** pro-environmental behavior, necessary condition analysis, power distance, nostalgia, awe of nature, COVID-19 risk perception

## Abstract

**Introduction:**

COVID-19 has altered human cognition and changed the cultural values of society. However, there has not been much debate among scholars about whether these above changes have led to an increase in pro-environmental behavior (PEB) of Chinese consumers.

**Methods:**

A comprehensive model was developed based on affective event theory. An online questionnaire was distributed, and 501 usable questionnaires were collected. In addition, two complementary approaches were employed: partial least squares structural equation modeling (PLS-SEM) and necessary condition analysis (NCA).

**Results:**

The PLS-SEM results showed that COVID-19 risk perception, nostalgia, the awe of nature, and attitude were found to have a positive effect on PEB; and the moderating effect of power distance belief (PD) between nostalgia (NO), attitude (AT) and PEB was confirmed. According to the NCA results, AT and NO are necessary conditions for the PEB of consumers.

**Discussion:**

This study provides deeper insight into the understanding of consumers’ pro-environmental behavior in the context of COVID-19 through the combined use of PLS-SEM and NCA.

## 1 Introduction

Urbanization and industrialization have had many negative impacts on ecosystems and human health, such as global warming, air pollution, urban waste, and loss of biodiversity, primarily due to the imbalance between human social development and ecological governance (Rosenmann et al., 2016; Alzubaidi et al., 2021). As a result of this unsustainable development mode combined with the destruction of the environment, a plague pandemic was eventually released that threatened public health as well as environmental safety around the globe (Daryanto et al., 2022; Zebardast and Radaei, 2022). Some scholars believe that COVID-19 is the revenge of nature due to the overacquisition of nature and the consumption of wild animals by humans (Cave and Dredge, 2020; Li et al., 2020; Liu et al., 2021). Global economies and societies have been impacted greatly by the COVID-19 crisis (Severo et al., 2021a; Milfont et al., 2022a). For example, in China, a widespread lockdown of cities, restrictions on public transportation and personal movement have been imposed by the Chinese government to prevent the spread of COVID-19 (Chen et al., 2020). Nevertheless, COVID-19 is also a turning point in promoting public awareness of the relationship between climate change, health, and sustainable living, as well as accelerating sustainable consumption (Ramkissoon, 2020; Tchetchik et al., 2021; Milfont et al., 2022b). Actually, a number of changes have taken place in the way consumers live and think due to the outbreak of COVID-19 (Büssing et al., 2021; Sun et al., 2022; Yang et al., 2022). As environmental degradation continues to worsen, an increasing number of consumers are becoming aware that individual behavior has a substantial impact on environmental problems (Schwartz et al., 2020; Moon et al., 2021). In addition, it is becoming increasingly common for people to reflect on and re-evaluate their relationship with the environment (Shakil et al., 2020; Mi et al., 2021). Therefore, it is crucial to explore the key factors influencing consumers’ pro-environmental behavior in the context of COVID-19.

**FIGURE 1:**
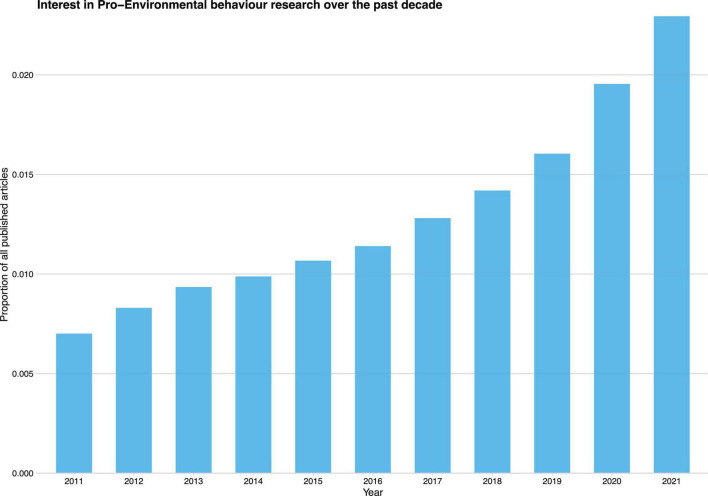
Interest in pro-environmental behavior research over the past decade.

Over the last decade, scholars have become increasingly interested in consumers’ pro-environmental behavior (Figure 1). A large and growing body of literature has investigated the antecedents of pro-environmental behavior, such as attitude (Alzubaidi et al., 2021; Sun et al., 2022), personal motivation (Yang et al., 2022), environmental knowledge and awareness (Chen et al., 2022), social and personal norms (Pearce et al., 2022), personality and environmental values (Simpson et al., 2021), self-efficacy and self-identification (Huang et al., 2022; Sharma et al., 2022b). Although scholars have provided explanations and insights based on a variety of theories and perspectives, some areas of research remain to be explored. First, a majority of previous studies regarding pro-environmental behavior have concentrated on individual psychological factors; however, these psychological factors may not be as important in predicting actual pro-environmental behavior as previously thought (Nielsen et al., 2022). Therefore, to promote pro-environmental behavior, it may be necessary to look beyond individual motivations to uncover some unexplained differences in attitude-behavior models by gaining a deeper understanding of how external factors influence behavior (Sun et al., 2019; Linder et al., 2021), for example, COVID-19.

Second, there has been a surge of interest in the impact of COVID-19 on pro-environmental behavior recently; however, the relationship between COVID-19 risk perception and pro-environmental behavior is not clear, and the results of some previous studies have been contradictory (Mi et al., 2021; Urban and Braun Kohlova, 2022). The COVID-19 pandemic presents a significant threat to humanity and raises issues about the ecological ethics of humans (Zebardast and Radaei, 2022). During the postpandemic era, people live in the shadow of the pandemic and continue to experience the various negative effects of the pandemic. The social cognition and behavior of individuals may be significantly altered by major external environmental events such as COVID-19 (Chen, 2020b; Zebardast and Radaei, 2022), and it has been found that people’s behavior varies considerably in different contexts (Wu et al., 2021). Therefore, it is necessary to further understand the relationship between the cognitive response to COVID-19 (i.e., COVID-19 risk perception) and consumer pro-environmental behavior from a new theoretical perspective.

Third, there is a relatively small body of literature that is concerned with the relationship between COVID-19 risk perception and consumer sentiment, such as nostalgia, environmental guilt, and awe of nature (Mi et al., 2021; Sun et al., 2021a; Kim et al., 2022). Individuals will not always act rationally (Koenig-Lewis et al., 2014; Sun et al., 2022); as a result, pro-environmental behavior is not always the result of a reasonable thought process or response to the environment (Gezhi and Xiang, 2022). Irrational factors such as emotions are ignored, which are to some extent more important than rational cognitive factors (Koenig-Lewis et al., 2014; Sun et al., 2021b). According to affective event theory, significant events in work scenarios can have an impact on employees’ emotions, and the positive and negative emotions generated by employees can have a significant impact on individuals’ attitudes and behaviors (Weiss and Cropanzano, 1996). Therefore, consumers’ behavior patterns may change when they are in the pandemic environment of COVID-19, considered a major external event that consumers have to contend with (Mi et al., 2021). As an important external factor, will people’s experiences and perceptions of the risks caused by COVID-19 prompt a profound reflection on existing eco-ethical issues such as the relationship between humans and nature, as well as a further improvement in the practice of pro-environmental behavior? Surprisingly, there has been insufficient attention given to this issue by researchers (Chi, 2021; Urban and Braun Kohlova, 2022).

To fill the aforementioned research gaps, three research questions were focused on. **RQ1**. What is the effect of COVID-19 risk perception on consumers’ emotions (i.e., nostalgia, guilt, and awe) and on attitude and pro-environmental behavior? **RQ2.** How does power distance belief (PD) moderate the relationships between emotions (i.e., nostalgia, awe), attitude, and pro-environmental behavior? **RQ3.** What is the necessary factor of the pro-environmental behavior of consumers in the context of the COVID-19 pandemic? To solve these research questions and obtain a more comprehensive understanding of the causal relationship between the variables, this study uses two complementary research methods to explore the mechanisms influencing consumer pro-environmental behavior from the dual perspective of adequacy (PLS-SEM) and necessity (NCA). In addition, a rigorous model was constructed based on affective event theory. Affective event theory, derived from organizational behavior, focuses on the emotional impact of work events on employees, whose positive and negative emotions, in turn, influence their attitudes and behaviors. Analogous to employees’ work events, we treat COVID-19 as a major external shock faced by consumers and explore the cognitive and emotional responses to external environmental events and how these responses affect consumers’ attitudes and behaviors. This study contributes to existing knowledge in terms of developing pro-environmental behavior research within the context of affective event theory and in light of the perception of risk associated with COVID-19. In particular, we examine how consumers’ cognitive response to external event shocks (COVID-19 risk perception) affects three consumer emotions (guilt, awe of nature, and nostalgia), which influence their attitudes toward environmental protection and pro-environmental behaviors. In addition, we further narrowed the attitude-behavior gap in the current pro-environmental behavior study by adding a cultural contextual factor (power distance).

## 2 Literature review and hypothesis

### 2.1 Pro-environmental behavior

Any behavior that benefits the environment or that strives to harm the environment as little as possible without causing significant harm to the environment is considered pro-environmental behavior (Kollmuss and Agyeman, 2002). In summary, a wide range of factors have been examined in the past to explore the factors influencing pro-environmental behavior, including sociodemographic characteristics, rational cognitive factors, and external situational factors. Scholars have focused on demographics in terms of gender (Sreen et al., 2018; Li et al., 2019), age (Wiernik et al., 2013), educational level (Gifford and Nilsson, 2014; Meyer, 2015), and income level (Meyer, 2015; Lim and Moon, 2022). In regard to rational cognitive factors, attitudes, norms, motivation, beliefs, and values play an important role (Poortinga et al., 2004; Gilg et al., 2005; Polonsky et al., 2012; Casaló et al., 2019; Punzo et al., 2019; Chen, 2020a; Gu et al., 2020), and the majority of these studies are based on the Theory of Planned Behavior (TPB), the Normative Activation Model (NAM), and the Value-Belief-Normative Theory (VBN). Additionally, external situational factors include infrastructure, individual capabilities, availability of technical equipment and products, governmental enforcement, public media, culture of society, and economic conditions (Tanner, 1999; Corraliza and Berenguer, 2000; Haddad, 2015; Sun et al., 2018; Shi et al., 2019; Zhou et al., 2019; He and Filimonau, 2020; Kaiser, 2021).

### 2.2 COVID-19 risk perception

Risk perception is considered a value judgment related to the uncertainty caused by a particular risk (Tversky and Fox, 1995) and can be understood from both cognitive and affective perspectives. People’s perceptions of COVID-19 risk are influenced by the danger it poses to their lives and health, which in turn influences their coping strategies and behavioral changes (Shulman et al., 2022). In addition to having a significant impact on the normal functioning of society, COVID-19 has also had profound effects on human social cognition and emotions (Sun et al., 2021b; Lawrance et al., 2022).

COVID-19 has had a significant impact on the economy and lives of humans. The widespread lockdown triggered feelings of boredom and loneliness (Zhou et al., 2022), making people nostalgic for the past (Gibbs and Egermann, 2021; Xia et al., 2021). A sense of awe is associated with perceived grandeur, which means that the individual has encountered something shocking. Second, the feeling of awe triggers a need for adaptation, i.e., the individual’s existing mental structure is insufficient to comprehend the awe-inspiring object (Keltner and Haidt, 2003). Additionally, widespread social blockades and restrictions on social distance have altered the way people live and consume (Vázquez-Martínez et al., 2021), which is consistent with the two core characteristics of awe-inspiring emotions. In addition, human arrogance and excessive claims of nature have led to an imbalance in the relationship between humans and nature, which has resulted in the COVID-19 pandemic. Consumers will be upset when they see the destruction of the ecological environment and serious social consequences, believe that humans are responsible for it, and feel guilty for not taking action to improve the environment (Lawrance et al., 2022). To alleviate this sense of guilt, individuals may engage in compensatory behaviors, such as green purchasing and pro-environmental behavior.

Previous studies have indicated that information and sensitivity about global crises such as COVID-19 positively impact individuals’ knowledge, attitudes, perceived behavioral control, and motivation and provide a basis for encouraging environmentally responsible behavior (Sun et al., 2022; Zebardast and Radaei, 2022). In addition, according to protective motivation theory, consumers’ perception of COVID-19 risk makes individuals vulnerable and feel that their lives and health are threatened; this state, in turn, shifts an individual’s focus from materialism to pro-social values (Shulman et al., 2022). Consumers may be inclined to minimize social contact due to the perceived risk of COVID-19 (Im et al., 2021), which may result in a preference and a greater sense of connection with the natural environment. It is possible that individuals may be more inclined to act in an environmentally friendly manner as a result of this perceived connection (Tam, 2013). It has also been shown that perceptions of COVID-19 risk and vulnerability stimulate empathy in individuals, and the emotion of empathy is an important psychological factor in motivating pro-environmental behavior (Yin et al., 2021; Ienna et al., 2022). Therefore, it is hypothesized that:

**H1a:** COVID-19 risk perception has a positive and significant effect on nostalgia.

**H1b:** COVID-19 risk perception has a positive and significant effect on guilt.

**H1c:** COVID-19 risk perception has a positive and significant effect on the awe of nature.

**H1c:** COVID-19 risk perception has a positive and significant effect on pro-environmental behavior.

### 2.3 Nostalgia, guilt, and awe of nature

The emotion of nostalgia serves key psychological functions as a positive, self-relevant, and social emotion (Sedikides et al., 2008). Nostalgia is a psychological variable that stimulates emotions and cognition; it can trigger positive actions or behavioral tendencies; and it can enhance social connection, facilitate social interaction, and serve as a catalyst for social interaction (Srivastava et al., 2022). The experience of nostalgia is often associated with positive emotions, allowing people to mentally escape the fast pace of modern life and return to an earlier time for solace. Temporary escapes provide psychological buffers and promote positive emotional and social relationships. In addition to providing a sense of presence, it enhances the sense that one is living a meaningful life (Zhang et al., 2021) and helps increase social connectedness and promote pro-social behavior (Christou et al., 2018). In addition to expressing the inner self through nostalgia, consumers become concerned about the welfare of others and develop greater empathy, while focusing on the inner self predicts more altruistic behaviors, including pro-environmental behaviors (Newman et al., 2014). However, few studies have explored the role of nostalgia in consumer pro-environmental behavior. Zhang et al. (2021) found that the emotion of nostalgia creates a sense of meaning in life, which in turn encourages consumers to invest in more sustainable recycling practices. Similarly, Wang et al. (2020) found that consumers’ product disposal behavior is affected differently by individual and collective nostalgia because they trigger different mechanisms.

Typically, guilt is a negative experience that occurs when an individual engages in moral reflection and takes responsibility for his or her actions after having hurt others, which plays an important role in prosocial behavior (Eisenberg et al., 2005; Teper et al., 2015). Compensatory ethics theory suggests that when an individual commits an unethical act, he or she will experience a strong sense of discomfort, which is caused by guilt (Zhong et al., 2010). The emotion of guilt plays an important role in influencing the attitude and behavior of the pro-environment (Escadas et al., 2019), and researchers have also explored how guilt affects individuals’ pro-environmental behavior as a type of pro-social behavior. Bamberg and Möser (2007) concluded, from a meta-analysis of 57 articles, that the feeling of guilt was one of the eight core variables that influence pro-environmental behavior. It is important to note that consumers’ feelings of guilt indicate that they have not achieved behavioral goals (e.g., environmentally friendly behaviors), which can drive motivation and change in attitudes toward environmentally friendly behaviors (Adams et al., 2020). It has also been shown in recent research that guilt plays a vital role in the change of attitudes toward environmental protection and the promotion of environmental behavior (Adams et al., 2020; Eom et al., 2021; Shipley and van Riper, 2021; Haj-Salem et al., 2022).

Awe is an emotional reaction to a stimulus that is perceived as powerful, vast, and beyond an individual’s cognitive abilities (Keltner and Haidt, 2003). This study defines awe as a self-transcending positive emotion that allows individuals to pay more attention to their surroundings than to themselves (Stellar et al., 2017; Zhao et al., 2018). There is evidence that awe leads to a number of positive effects on an individual. From a cognitive perspective, awe not only expands an individual’s perception of time’s abundance (Rudd et al., 2012) but can also lead to systematic cognitive processes. As a positive emotion of self-transcendence, awe enables individuals to extend their self-concept to pay more attention to their surroundings and outside world (Piff et al., 2015; Stellar et al., 2017; Zhao et al., 2018), and an individual’s pro-environmental behavior is stimulated when the natural environment is integrated into their self-concept (Schultz, 2001). By transforming the notion that humans are not above nature (Wang et al., 2019) but instead are part of the natural world, individuals feel that they are not isolated, resulting in an inescapable sense of responsibility for existing environmental consequences, which in turn promotes individual pro-environmental behavior (Jacobs and McConnell, 2022). Individuals with this mindset tend to be more conscious of the consequences of their actions, re-examine the relationship between humans and nature (Wang et al., 2019), and change their attitudes toward nature. From an emotional perspective, awe can also bring spiritual pleasure to individuals (Rudd et al., 2012), thereby enhancing their prosocial behavior (Prade and Saroglou, 2016). Therefore, it is hypothesized that:

**H2a:** Nostalgia has a positive and significant effect on pro-environmental behavior.

**H2b:** Nostalgia has a positive and significant effect on attitude.

**H3:** Guilt has a positive and significant effect on attitude.

**H4a:** Awe of nature has a positive and significant effect on attitude.

**H4b:** Awe of nature has a positive and significant effect on pro-environmental behavior.

### 2.4 Attitude

A person’s attitude toward the environment has a significant impact on his or her behavior and intentions in relation to the environment (Cheung and To, 2019). Despite the fact that scholars acknowledge the discrepancy between consumers’ attitudes and actual behavior (Yadav et al., 2019), environmental attitude is still considered to be one of the best predictors of actual environmental behavior (Herbes et al., 2020; Felix et al., 2022). According to the theory of planned behavior (Ajzen, 1991), an individual’s behavior can be effectively controlled by his or her inner subjective consciousness and is a rational decision. An individual’s behavioral attitudes influence their behavioral intentions, which in turn influence their actual behavior. In terms of cognitive dissonance theory, people tend to act on their beliefs to avoid mental discomfort (Festinger, 1962). It appears that people with a higher level of environmental awareness demonstrate more pro-environmental behaviors (Lin et al., 2022). Therefore, it is hypothesized that:

**H5:** Attitude has a positive and significant effect on pro-environmental behavior.

### 2.5 Power distance belief

The concept of power distance is a cultural value that reflects the individual’s acceptance of the gap in power (Hofstede, 1984, 1989). In social cultures where power distance is generally high, vertical hierarchical relationships are strongly felt. As a result of accepting power differences, individuals are expected to act in accordance with their position in the social hierarchy and maintain the existing state of power distribution, and they may maintain this position in the social hierarchy. Compared to Westerners, Chinese people are heavily influenced by Confucian culture and tend to have a higher power distance orientation (Thompson et al., 2020). Due to the long-term influence of Confucian culture and the high-powered distance society, individuals view inequality as justified as a result of power distribution, and higher-ranking individuals will receive greater “rent-seeking,” resulting in an unequal distribution of income. As a result, Chinese consumers not only subconsciously accept their social class and position but also link their social responsibility to their social status. In light of the high cost of pro-environmental behavior (Wang and Chao, 2019; De Silva et al., 2021), many Chinese residents view pro-environmental behavior as a greater social responsibility that should be borne by external forces with more wealth and power, such as celebrities, large corporations, or the central government (Dendler and Dewick, 2016; De Silva et al., 2021).

Despite the fact that the Chinese have a high PD, there is a wide variation in the degree to which individuals accept unequal power distributions within institutions and organizations (Kirkman et al., 2009; Mi et al., 2020). As a result, individuals with high PDs are more sensitive to the concept of social status than those with low PDs (Kaynak et al., 2013). Previous research has indicated that emotions influence attitudes and behavior differently depending on cultural values (Onwezen et al., 2014). According to their study, both anticipated pride and guilt have an effect on environmental behavior; however, the extent of this effect varies between individuals in individualistic and collectivist countries (Onwezen et al., 2014). Therefore, the present study suggests that even individuals with the same attitudes and specific emotions may still exhibit inconsistent pro-environmental behaviors in different power distance contexts, implying that power distance moderates the relationship between attitudes and specific emotions and pro-environmental behaviors. As a result, it is hypothesized that:

**H6a:** Power distance weakens the relationship between nostalgia and pro-environmental behavior.

**H6b:** Power distance weakens the relationship between attitude and pro-environmental behavior.

**H6c:** Power distance weakens the relationship between awe of nature and pro-environmental behavior.

**H6d:** Power distance weakens the relationship between COVID-19 risk perception and pro-environmental behavior.

**FIGURE 2:**
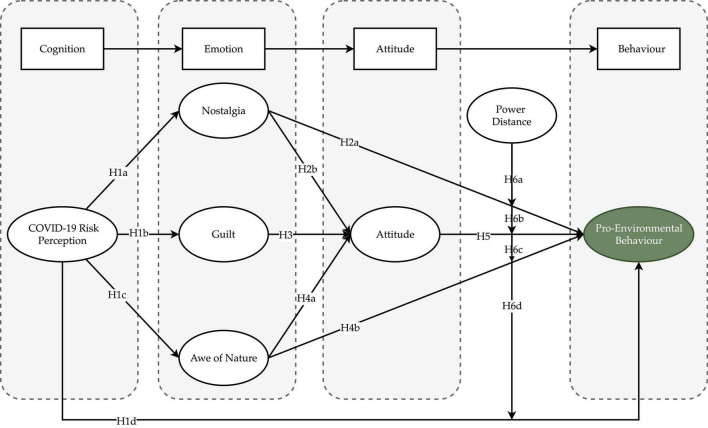
Conceptual framework.

In sum, based on affective event theory, we developed a rigorous model. The conceptual model in Figure 2 includes cognitive elements, emotional elements, and contextual cultural influences that impact consumer behaviors.

## 3 Data collection, measurements, and samples

Due to the social distance restrictions in China, face-to-face interviews should be avoided; thus, this research was conducted online. Referring to the data collection procedure of previous studies (Mi et al., 2021; Hua and Dong, 2022; Xiang et al., 2022), the pilot study and formal survey were conducted through “WJX,” a famous online platform in China. To protect the privacy of the participants and to mitigate the effects of social desirability bias, we stated in the questionnaire that the data collection was for academic research only, that the research was anonymous and that all information would be kept strictly confidential. To ensure the quality of the questionnaire, an honorarium of 5–10 RMB (roughly 0.7–1.6 dollars) was awarded to each participant who answered the questionnaire.

Prior to conducting the formal survey, a pilot study was conducted to ensure the reliability and validity of the measurement scale. A total of 55 questionnaires were distributed through the online platform, the feedback collected from the pre-study was summarized, and the semantics and expressions of some scales were modified and adjusted appropriately. After the deletion of some questions, all the scales in the pilot study passed the reliability and validity tests and formed the final questionnaire. Before the formal survey, a priori power analysis was conducted in G power 3.1.9.6 to determine an appropriate sample size. With a medium effect size of 0.15 (Ducoffe, 1995), an alpha of 0.05, and a power of 0.90, a sample size of 146 was needed for the following study. The formal study was conducted in April 2022 and lasted for 1 month. After filtering for straight lining and missing data, 44 responses were deleted from a total of 545 questionnaires. Finally, 501 valid questionnaires were collected. Compared with the required sample size in G power, our sample size was deemed appropriate. As convenience sampling is widely used in marketing, consumer behavior and social science research (Santos and Gonçalves, 2019; Gupta et al., 2020), it is considered acceptable considering population size, time, and cost to use the convenience sampling technique (Kapoor and Dwivedi, 2020).

A questionnaire was developed to evaluate the effects of COVID-19 on pro-environmental behaviors (Supplementary Appendix 7). The proposed conceptual model was tested using widely used and validated measurement items, including COVID-19 risk perception (O’Connor and Assaker, 2022), nostalgia (Wildschut et al., 2006), guilt (Ágoston et al., 2022), awe of nature (Shiota et al., 2006), attitude (Qin and Hsu, 2022), power distance (Yoo et al., 2011), and pro-environmental behavior (Stern, 2000). Research items were measured using a five-point Likert scale (1 being highly disagree and 5 being highly agree). In addition, four demographic variables, namely, gender, age, education level, and monthly income status (RMB), were selected as control variables. The demographic characteristics of the respondents are summarized in Table 1.

**TABLE 1:** Sociodemographic profile of respondents.

Measure	Item	Frequency	Percent	Cumulative percent
Gender	Male	226	45.1	45.1
	Female	275	54.9	100
Age	Below 20	32	6.4	6.4
	21–30	295	58.9	65.3
	31–40	131	26.1	91.4
	41–50	26	5.2	96.6
Edu	50 and over	17	3.4	100
	Junior high school or below	17	3.4	3.4
	Senior high school	56	11.2	14.6
	Technical college	103	20.6	35.1
	Junior college or university	259	51.7	86.8
	Master’s degree or PhD	66	13.2	100
Income/month (RMB)	Less than 3,500	178	35.5	35.5
	3,500–6,000	154	30.7	66.3
	6,001–8,000	94	18.8	85
	8,001–9,999	64	12.8	97.8
	over 10,000	11	2.2	100
	Total	501	100	

## 4 Results

The proposed model was tested using multiple methods. The normality of the data was assessed by using a one-sample Kolmogorov–Smirnov (KS) test. In Supplementary Appendix 4 and the QQ plot (Supplementary Appendix 5), it appears that the two-tailed *p*-values are below 0.05, which indicates that the distribution does not follow a normal distribution. We then used partial least squares structural equation modeling (PLS-SEM) to calculate the net effect of independent variables due to its resilience to non-normal distributions (Hair et al., 2019).

### 4.1 PLS-SEM result

#### 4.1.1 Common method bias

**FIGURE 3:**
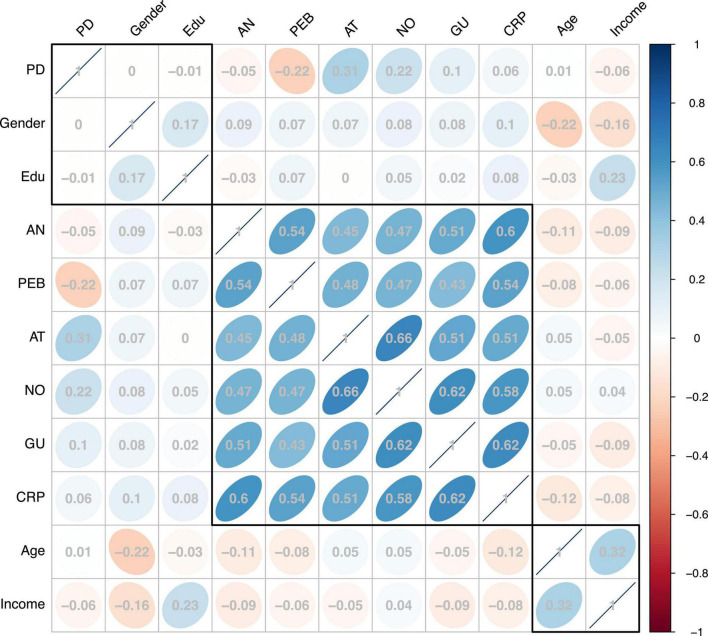
Correlation between variables.

To reduce the influence of common method bias (CMB) on the study results, the English scales extracted from foreign literature were back translated, and accurate language expressions were presented to the respondents through repeated comparisons and corrections, thus reducing the possibility of errors due to language expression ambiguity. In addition, as a statistical test, we conducted Harman’s single factor to determine whether CMB was present using SPSS 26, and seven factors in this study were combined to produce a single factor. The result showed that the newly formed factor explained 39.506% of the variation, which is less than the 50% requirement (Podsakoff et al., 2003; Leong et al., 2020b). In addition, to further confirm that CMB is not a problem, we calculated the substantive variance and the method variance by converting each item into a single-item second-order construct (Podsakoff et al., 2003). According to Table 2, the average substantively explained variance of the indicators is 0.881, while the variance based on the average method is 0.013, with most factor loadings not significant. Furthermore, the ratio 60:1 between the substantive variance and the method variance confirms the lack of concern regarding CMB (Leong et al., 2020a). Additionally, as shown in Figure 3, there was no particularly high correlation between the variables in this study (*r* > 0.9). This indicates that CMB does not pose a threat to our findings (Bagozzi et al., 1991). In summary, based on the analysis above, CMB is not an issue in this study.

**TABLE 2:** Common method bias analysis.

Construct	Indicator	Substantive factor loading (R1)	*T*-value	R1^2^	Method factor loading (R2)	*T*-value	R2^2^
AN	AN1	0.884	41.001	0.781	0.041	1.402	0.002
	AN2	0.933	41.108	0.870	–0.045	1.512	0.002
	AN3	0.893	33.858	0.797	0.003	0.092	0.000
AT	AT1	0.853	36.701	0.728	0.043	1.446	0.002
	AT2	0.816	30.295	0.666	0.077	2.346	0.006
	AT3	0.963	38.391	0.927	–0.125	3.769	0.016
CRP	CRP1	0.660	15.519	0.436	0.247	5.479	0.061
	CRP2	0.675	17.152	0.456	0.210	5.222	0.044
	CRP3	0.767	17.816	0.588	0.129	2.716	0.017
	CRP4	1.104	30.405	1.219	–0.266	6.183	0.071
	CRP5	1.087	26.892	1.182	–0.264	5.630	0.070
	CRP6	0.894	16.350	0.799	–0.096	1.622	0.009
GU	GU1	0.801	29.619	0.642	0.110	3.546	0.012
	GU2	0.882	27.097	0.778	–0.060	1.527	0.004
	GU3	0.861	29.861	0.741	0.034	1.010	0.001
	GU4	0.952	34.383	0.906	–0.091	2.648	0.008
NO	NO1	0.878	33.214	0.771	0.035	1.080	0.001
	NO2	0.925	35.503	0.856	–0.039	1.197	0.002
	NO3	0.902	35.308	0.814	0.003	0.094	0.000
PEB	PEB1	0.820	33.334	0.672	0.115	3.716	0.013
	PEB2	0.840	32.268	0.706	0.047	1.451	0.002
	PEB3	0.842	28.926	0.709	0.042	1.211	0.002
	PEB4	0.917	31.536	0.841	–0.108	3.178	0.012
	PEB5	0.927	27.160	0.859	–0.111	2.861	0.012
PD	PD1	0.908	125.277	0.824	0.027	1.462	0.001
	PD2	0.869	66.731	0.755	0.016	0.766	0.000
	PD3	0.911	98.184	0.830	–0.037	2.076	0.001
	PD4	0.903	99.005	0.815	–0.027	1.334	0.001
	PD5	0.888	78.078	0.789	0.022	1.073	0.000
**Average**		**0.881**		**0.785**	−**0.002**		**0.013**
**Ratio**	**60**						

AN, awe of nature; AT, attitude; CRP, COVID-19 risk perception; GU, guilt; NO, nostalgia; PD, power distance; PEB, pro-environmental behavior. Bold values are the average value of substantive factor loading and method factor loading and the ratio of them.

#### 4.1.2 Assessment of the measurement model

The PLS-SEM measurement model is evaluated based on three criteria: reliability, convergent validity, and discriminant validity (Fornell and Larcker, 1981). According to Hair et al. (2021), Cronbach’s alpha, composite reliability (CR), and rho_A can be used to measure internal consistency. All three values exceeded the accepted cut-off point of 0.7 (Hair et al., 2014), demonstrating the reliability of the scale (Table 3). In assessing the convergent validity of a measurement model, researchers can use the outer loading (>0.708) and the average variance extracted (AVE) scores (>0.5) of each observed item (Fornell and Larcker, 1981; Bagozzi et al., 1991). Table 3 shows that all items exceeded the acceptable value, indicating the study had convergent validity.

**TABLE 3:** Assessment of the reliability and convergent validity.

Construct	Item	Loading	Cronbach’s alpha	rho_A	CR	AVE
AN	AN1	0.917	0.887	0.889	0.93	0.816
	AN2	0.899				
	AN3	0.894				
AT	AT1	0.886	0.848	0.855	0.908	0.766
	AT2	0.88				
	AT3	0.86				
CRP	CRP1	0.884	0.929	0.937	0.944	0.737
	CRP2	0.868				
	CRP3	0.885				
	CRP4	0.863				
	CRP5	0.846				
	CRP6	0.801				
GU	GU1	0.892	0.896	0.899	0.928	0.762
	GU2	0.831				
	GU3	0.89				
	GU4	0.877				
NO	NO1	0.907	0.885	0.886	0.929	0.812
	NO2	0.891				
	NO3	0.906				
PD	PD1	0.9	0.939	0.946	0.953	0.802
	PD2	0.863				
	PD3	0.915				
	PD4	0.908				
	PD5	0.892				
PEB	PEB1	0.907	0.917	0.92	0.938	0.752
	PEB2	0.878				
	PEB3	0.874				
	PEB4	0.835				
	PEB5	0.842				

AN, awe of nature; AT, attitude; CRP, COVID-19 risk perception; GU, guilt; NO, nostalgia; PD, power distance; PEB, pro-environmental behavior.

To assess the discriminant validity, two types of assessments were conducted: the Fornell-Larcker criterion (Fornell and Larcker, 1981) and heterotrait-monotrait ratios (Henseler et al., 2015). The Fornell-Larcker criterion examines the correlation coefficient between the square root of the mean variance extraction and other latent variables of the measurement model. To satisfy the Fornell-Larcker criteria, the AVE of one latent variable must be larger than the squared correlation with the other latent variable (Fornell and Larcker, 1981). Table 4 shows that the criteria have been met. The HTMT value is calculated by comparing the mean cross-correlation coefficient between observed variables of different latent profiles to the mean correlation coefficient between observed variables of the same latent profile. Discriminant validity is established if the value of HTMT is less than 0.85 (Henseler et al., 2014) or 0.90 (Carrión et al., 2017). As seen in Table 4, all obtained values are below 0.85, demonstrating good discriminant validity.

**TABLE 4:** Assessment of the discriminant validity.

	AN	AT	CRP	GU	NO	PD	PEB
**Fornell-Larcker criterion**							
AN	**0.903**						
AT	0.450	**0.875**					
CRP	0.604	0.514	**0.858**				
GU	0.510	0.520	0.631	**0.873**			
NO	0.470	0.665	0.588	0.618	**0.901**		
PD	−0.048	0.309	0.050	0.101	0.215	**0.896**	
PEB	0.535	0.482	0.542	0.435	0.468	−0.222	**0.867**
**HTMT**							
AN							
AT	0.516						
CRP	0.655	0.571					
GU	0.570	0.592	0.681				
NO	0.530	0.763	0.642	0.693			
PD	0.057	0.349	0.075	0.111	0.237		
PEB	0.592	0.540	0.575	0.473	0.516	0.240	

The off-diagonal values (bold) in the above matrix are the square correlations between the latent constructs and the diagonals are AVEs.

#### 4.1.3 Assessment of the structural model

**FIGURE 4:**
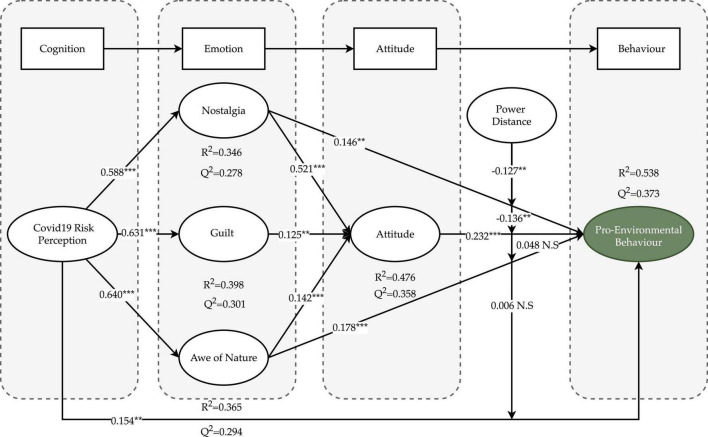
Inner model result of PLS-SEM. ***p < 0.001, **p < 0.01.

Initially, the structural model was examined for collinearity by examining the variance inflation factors (VIFs) of all predictor constructs. In Table 5, it is evident that all variables’ VIF values were less than 3 (1.188–2.528), indicating that there was no issue with collinearity (Hair et al., 2019; Hair et al., 2021). Next, bootstrapping was employed to test the significance of the hypothesis with 5,000 subsamples (Hair et al., 2021). As shown in Figure 4 and Table 5, the results showed that the effects of COVID-19 risk perception on nostalgia (β = 0.588, *t*-value = 16.958), moral anger (β = 0.631, *t*-value = 19.992), and awe of nature (β = 0.604, *t*-value = 20.004) were all significant, thereby supporting H1a, H1b, and H1c. In addition, nostalgia (β = 0.521, *t*-value = 12.284), moral anger (β = 0.125, *t*-value = 2.805), and awe of nature (β = 0.142, *t*-value = 3.631) demonstrated a significant positive effect on attitude, supporting H2b, H3, and H4a, respectively. Additionally, COVID-19 risk perception (β = 0.154, *t*-value = 3.284), nostalgia (β = 0.146, *t*-value = 2.905), awe of nature (β = 0.178, *t*-value = 4.214), and attitude (β = 0.232, *t*-value = 4.550) were found to have a positive effect on pro-environmental behavior, thus supporting H1d, H2a, H4b, and H5.

**TABLE 5:** Assessment of structural model.

Hypothesis	Coefficient	Std	*T*-values	*P*-values	*f* ^2^	VIF	Result
CRP - > NO	0.588	0.035	16.958	***	0.530		Supported
**NO; *R*^2^ = 0.346; *Q*^2^ predict = 0.278**							
CRP - > GU	0.631	0.032	19.992	***	0.662		Supported
**GU; *R*^2^ = 0.398; *Q*^2^ predict = 0.301**							
CRP - > AN	0.604	0.03	20.004	***	0.574		Supported
**AN; *R*^2^ = 0.365; Q^2^ predict = 0.294**							
NO - > AT	0.521	0.042	12.284	***	0.304	1.707	Supported
GU - > AT	0.125	0.045	2.805	**	0.017	1.798	Supported
AN - > AT	0.142	0.039	3.631	***	0.027	1.425	Supported
**AT; *R*^2^ = 0.476; *Q*^2^ predict = 0.358**							
AN - > PEB	0.178	0.042	4.214	***	0.039	1.775	Supported
AT - > PEB	0.232	0.051	4.55	***	0.046	2.528	Supported
CRP - > PEB	0.154	0.047	3.284	**	0.022	2.306	Supported
NO - > PEB	0.146	0.05	2.905	**	0.019	2.412	Supported
PD - > PEB	−0.329	0.036	9.146	***	0.197	1.188	Supported
**PEB; *R*^2^ = 0.538 *Q*^2^ predict = 0.373**							
PD × AN - > PEB	0.048	0.039	1.228	0.220			Not supported
PD × NO - > PEB	−0.127	0.047	2.719	**			Supported
PD × CRP - > PEB	0.006	0.044	0.139	0.889			Not supported
PD × AT - > PEB	−0.136	0.044	3.103	**			Supported

***p < 0.001, **p < 0.01.

**FIGURE 5:**
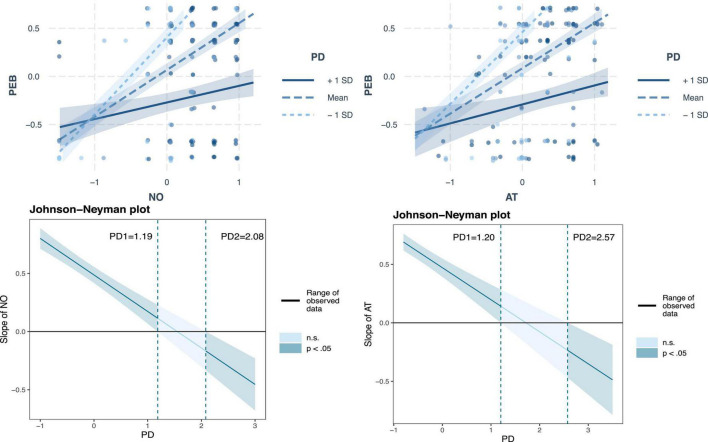
Moderating effect of power distance belief.

In addition, a two-stage approach was used to examine the moderation effect using Smart-PLS (Henseler and Fassott, 2010). The results of the moderating effect test in Table 5 showed that power distance (PD) had a significant negative effect on the relationship between nostalgia and pro-environmental behavior (β = −0.1127, *t*-value = 2.719) and attitude and pro-environmental behavior (β = −0.136, *t*-value = 3.103), indicating that different levels of power distance negatively impact the relationship between nostalgia and pro-environmental attitudes. Thus, H6a and H6b were supported. Unexpectedly, the interaction effects between awe of nature and power distance (β = 0.048, *t*-value = 1.228) and COVID-19 risk perception and power distance (β = 0.006, *t*-value = 0.139) on pro-environmental behavior were not significant. Therefore, H6c and H6d were not supported. Additionally, the Johnson–Neyman technique was employed to further understand the moderating effect of power distance (Spiller et al., 2013). This method identifies the level from which the moderating variable has a moderating effect on the relationship between the independent and dependent variables. As shown in Figure 5, the moderating effect of power distance on the relationship between nostalgia and pro-environmental behavior is not significant when PD is below 1.19 and above 2.08. Similarly, the moderating effect of power distance on the relationship between attitude and pro-environmental behavior is not significant when PD is below 1.20 and above 2.57.

Next, the coefficient of determination (*R*^2^) was used to evaluate the explained variance. According to Chin et al. (2003), the *R*^2^-value is considered small (0.19), moderate (0.33), and substantial (0.67). In Table 5, COVID-19 risk perception explained 34.6, 39.8, and 36.5% of the variation in nostalgia, moral outrage, and awe of nature, respectively, demonstrating significant explanatory power. In addition, to establish predictive criteria, we calculated effect sizes using prognostic relevance in Stone-Geisser *Q*^2^. The *Q*^2^-values were 0.278 for nostalgia, 0.301 for moral anger, 0.294 for awe of nature, 0.358 for attitude, 0.373 for pro-environmental behavior, all of which were above zero (Hair et al., 2013). In terms of effect size *f*^2^, which measures the substantial influence of an exogenous variable, a value of 0.02, 0.15, and 0.35 represents a small, medium, and large effect of the corresponding exogenous variable (Cohen, 1988). As seen in Table 5, COVID-19 risk perception had a substantial effect on nostalgia (*f*^2^ = 0.530), moral anger (*f*^2^ = 0.662), and awe of nature (*f*^2^ = 0.574). Nostalgia and power distance had a moderate effect on attitude (*f*^2^ = 0.304) and pro-environmental behavior (*f*^2^ = 0.197), respectively.

Regarding the model fit of this study, both the standardized root mean square residual (SRMR) and goodness of fit (GOF) were used. The SRMR value was 0.050, which was below the recommended maximum of 0.08 (Henseler et al., 2016), indicating a good overall fitness of the proposed framework. The GOF is defined as the geometric mean of the extracted variance and the average of the *R*^2^ of all endogenous variables, and the GOF value of the research framework was calculated as follows.


$$G\,O\,F=\sqrt{\bar{A\,V\,E}\times \bar{R^{2}}}=\sqrt{0.425\times 0.778}=0.575$$

As suggested by Wetzels et al. (2009), the GOF value was small (0.1), medium (0.25), and large (0.36). The GOF for this study is 0.575, which is larger than the recommended value. Taking into consideration both SRMR (0.050) and GOF values (0.575), it can be concluded that the research model in this study is appropriate.

### 4.2 Necessary condition analysis (NCA) result

NCA is a new method of necessary condition analysis (NCA) based on complex causality; it not only identifies the necessary conditions of the outcome variables but also calculates the effect size and bottlenecks of these conditions quantitatively. It is used to determine the size of the “necessary-but-not-sufficient-condition” effects between independent variables and dependent variables (Dul, 2016). As a complement to traditional adequacy analysis techniques, NCA quantifies the number of antecedent conditions required for achieving a particular level of outcome variables by analyzing the effect size and bottleneck of antecedent conditions (Dul et al., 2020).

**FIGURE 6:**
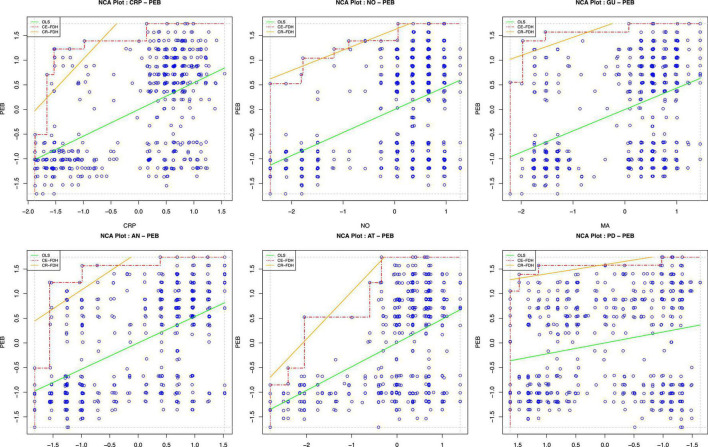
Scatter plots of necessary condition analysis.

**FIGURE 7:**
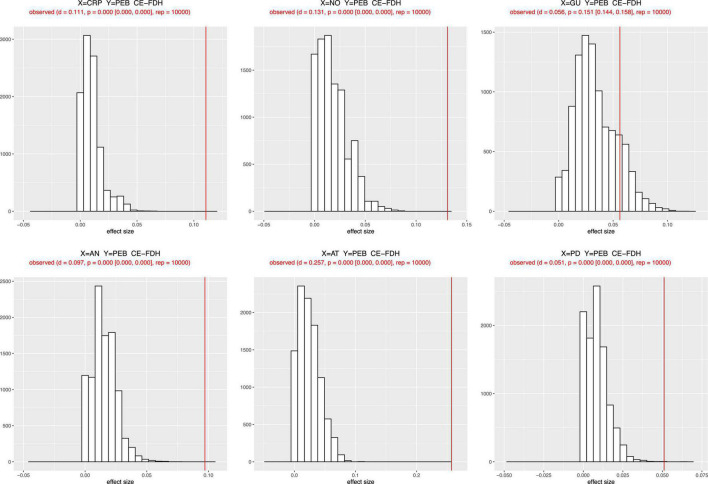
Histograms with bottleneck values.

First, to conduct the NCA, latent variable scores were obtained using the PLS-SEM procedure (Richter et al., 2020), and then the NCA package in R was employed to perform NCA analysis (Dul et al., 2018). As a starting point, an NCA consists of drawing a ceiling line through the upper-left observations of an x-y plot, and the scatter plots for all relevant relations are shown in Figure 6. Second, using a recommended random sample size of 10,000, we tested the statistical significance of the effect sizes (d) of the latent variable scores (Dul, 2016; Dul et al., 2020). Since the CE-FDH line is appropriate for survey data rated on a five-point Likert scale, we interpreted the NCA results using its parameters (Vis and Dul, 2018). The results in Figure 7 and Table 6 meaningfully (*d* ≥ 0.1) and significantly (*p* < 0.05) reveal that AT (*d* = 0.257, *p* < 0.001) and NO (*d* = 0.131, *p* < 0.001) are necessary conditions for the pro-environmental behaviors of consumers. Finally, the bottleneck technique was used to assist in specifying threshold levels for achieving a particular level of performance. As shown in Table 7, to achieve a low level of pro-environmental behavior (30%), AT (9.4%), CRP (0.2%) are necessary; to have a medium level of PEB (70%), AN (7.9%), AT (52.4%), CRP (6.5%), GU (6.6%), and NO (16.4%) are necessary. However, at a high level of pro-environmental behavior (100%), six necessary conditions should be present: AN at no less than 66.1%, AT at no less than 58.6%, CRP at no less than 58.9%, GU at no less than 62.4%, NO at no less than 67.3%, and PD at no more than 20.2%.

**TABLE 6:** Necessary condition analysis result (Method: CE-FDH).

Construct	Ceiling zone	Scope	Effect size (d)	*P*-values	Conditional inefficiency (%)	Outcome inefficiency (%)
**PEB**						
AN	1.123	11.557	0.097	***	33.898	34.794
AT	3.728	14.495	0.257	***	41.455	24.868
CRP	1.313	11.868	0.111	***	41.058	20.114
GU	0.563	15.381	0.056	0.154	37.567	65.546
NO	1.667	12.728	0.131	***	32.748	64.734
PD	0.576	11.274	0.051	***	20.239	80.070
**AT**						
AN	0.403	14.058	0.029	0.006	82.887	75
GU	0.563	15.381	0.037	0.064	87.339	59.433
NO	0.455	15.482	0.029	0.047	75	83.545

***p < 0.001.

**TABLE 7:** Bottleneck table (percentages).

PEB	AN	AT	CRP	GU	NO	PD
0	NN	NN	NN	NN	NN	NN
10	NN	NN	NN	NN	NN	NN
20	NN	NN	NN	NN	NN	NN
30	NN	9.4	0.2	NN	NN	NN
40	7.9	18.0	6.5	NN	NN	NN
50	7.9	18.0	6.5	NN	NN	NN
60	7.9	18.0	6.5	NN	NN	NN
70	7.9	52.4	6.5	6.6	16.4	NN
80	7.9	52.4	10.2	6.6	33.6	NN
90	25.0	58.6	58.9	18.5	50.0	85.1
100	66.1	58.6	58.9	62.4	67.3	20.2

NN, not necessary.

## 5 Discussion and conclusion

### 5.1 Theoretical implications

Overall, the majority of previous studies have examined the impact of rationality, affection, and culture separately on consumers’ pro-environmental behavior, while few have examined their combined effect. This study makes the following contributions.

First, drawing on affective event theory, this study constructs a comprehensive model that explores how external event shocks (COVID-19) affect consumers’ pro-environmental behavior. To our knowledge, this is one of the few studies that use affective event theory to investigate consumers’ pro-environmental behavior, expanding the perspective for understanding consumer behavior.

Second, an increasing number of scholars argue that cognitive and affective factors should be integrated into a unified rational and emotional theory of environmental behavior to better explain individuals’ pro-environmental behavior (Tian and Liu, 2022). Unfortunately, a limited number of studies have explored the interaction between emotional and cognitive factors. As a result, we intend to further investigate the interaction between rational and emotional factors in our study, as cognitive and emotional factors are mutually influential (Luo and Chea, 2018).

Third, the majority of previous studies focusing on the pro-environmental behavior of consumers used first-generation multivariate methods (such as multiple regression analysis) and second-generation multivariate methods [such as structural equation modeling (SEM)], which assume symmetrical relations between variables; these studies primarily explored the net effect of antecedent variables. However, using PLS-SEM and NCA, this study provides further insight into the mechanisms influencing pro-environmental behavior from the dual perspectives of adequacy and necessity.

Finally, whether consumers’ attitudes translate into actual behaviors depends on the specific context in which they live (Zhang et al., 2019; Sun et al., 2022). Most studies of people’s behavioral and psychological reactions to the outbreak of COVID-19 have been based in Western countries where individualism is prevalent, and relatively few studies have been conducted in countries where collectivism and power distance are high (Kwon and Park, 2022). Thus, this study explores the pro-environmental behaviors of consumers with different PDs during the COVID-19 pandemic in China and broadens the boundary conditions for understanding pro-environmental behaviors.

### 5.2 Discussion of findings

In this study, we extend the body of knowledge on pro-environmental behavior during the COVID-19 pandemic. The purpose of this study is to contribute to the theoretical understanding of consumer behavior concerning environmental issues and to provide insight into how to develop ecological environments in a sustainable manner.

First, according to the PLS-SEM results, COVID-19 risk perception had a significant effect on pro-environmental behavior and specific emotions, which is consistent with previous studies. Consumers’ perception of COVID-19 risk creates a sense of crisis, which significantly influences people’s pro-environmental behavior (Maleksaeidi and Keshavarz, 2019; Kim et al., 2022). Due to COVID-19, people are now more environmentally conscious in their consumption, believing that if we do not care for the environment, pandemics and disasters will happen again in the future (Lucarelli et al., 2020). However, the conclusion in this study is different from that of Urban and Braun Kohlová (2022), who found that there was no impact of the COVID-19 crisis on environmental attitudes or green decisions. The possible reason for the inconsistency in research is that previous studies ignored the role of individual emotions. Alternatively, only when a certain emotion is evoked in the consumer will he or she actually engage in pro-environmental behavior. As mentioned earlier, among the factors influencing consumers’ pro-environmental behavior, emotional factors can play a more decisive role than cognitive factors in some cases. According to affective events theory, it is important to recognize that an individual’s behavior is not always dictated by his or her rational perceptions. External events in life can cause negative or positive emotional reactions, and these emotional reactions can have a significant impact on attitudes and behaviors.

Second, in terms of emotions, the PLS-SEM results revealed that nostalgia had a significant effect on attitude and pro-environmental behavior, which is consistent with previous studies. Wang et al. (2020) and Zhang et al. (2021) found that the emotion of nostalgia had a positive effect on consumers’ pro-environmental behaviors, such as sustainable recycling and product disposal. However, Wang and Chao (2019) found that green products are less preferred by consumers with a high feeling of nostalgia; consumers with a strong sense of nostalgia tend to dwell on the past, which inhibits their preference for green products, as green products are often associated with the future (Alzubaidi et al., 2021). This inconsistency in research occurs because nostalgia is a complex concept, and different types of nostalgia have different effects on consumer behavior (Srivastava et al., 2022). According to Boym (2008), there are two types of nostalgia: “restorative nostalgia” and “reflective nostalgia.” “Reflective nostalgia” refers to reflecting on the remembered past for the purpose of the present and is often associated with establishing continuity. This kind of nostalgia is a positive experience that provides individuals with the opportunity to reflect on the past, which enhances self-worth, self-esteem, and social connectedness and promotes pro-social behavior (Srivastava et al., 2022; Zhang and Tao, 2022). There may be a tendency for individuals to invoke nostalgia as a coping mechanism to deal with the social distance restrictions of COVID-19 and the resulting isolation and social disconnection. This may be done as a means to regain a sense of self-continuity and meaning (Xia et al., 2021). We believe that the nostalgia triggered by COVID-19 is a “reflective nostalgia,” which promotes pro-environmental behavior. Additionally, according to the NCA result, nostalgia (*d* = 0.131, *p* < 0.001) was a necessary condition for pro-environmental behavior.

In addition, the emotion of guilt was found to have a significant effect on pro-environmental behavior, which is consistent with previous studies. Increasingly, consumers are becoming aware that COVID-19 outbreaks are linked to unsustainable consumption patterns. When people realize they could have avoided it, they develop negative guilt (Rees et al., 2015), which may cause them to take responsibility for the environmental impact (Daryanto et al., 2022). An ingrained sense of personal responsibility and willingness to act morally compelled to mitigate COVID-19 triggered by guilt can also contribute to the promotion of actions that are environmentally responsible (Milfont et al., 2022b). Similarly, an awe of nature was found to have a positive and significant effect on attitude and pro-environmental behavior. An individual is more inclined to be generous (Yang and Hu, 2021), to engage in helping activities (Wu et al., 2022) and to devote more time to charitable activities when instilled with the feeling of awe (Piff et al., 2015). A previous study also found that awe significantly influences pro-environmental behavior as a manifestation of pro-social behavior (Ramus and Killmer, 2007).

Additionally, the moderating effects of PD were confirmed. PD was found to have a significant moderating effect between nostalgia and pro-environmental behavior, which echoes the inconsistency of findings from previous studies regarding nostalgia and pro-environmental behavior (Wang and Chao, 2019; Wang et al., 2020; Zhang et al., 2021). This result confirms that the influence of consumers’ specific emotions on pro-environmental behavior varies across cultural contexts. As indicated in previous studies, the perception of climate change by individuals is often closely related to their personal values and worldviews; and cultural orientation can have a significant impact on their environmental attitudes and behaviors (Price et al., 2014). Due to the inequitable distribution of social power, consumers with high PDs take their ignorance of pro-environmental behaviors for granted and justify it with a vengeance. In contrast, individuals in low power distance cultures believe that pro-environmental behavior is one of their responsibilities and obligations and that pro-environmental behavior is dependent upon their participation. Previous research has shown that Chinese consumers have a strong reliance on the government for pro-environmental activities (Chan, 2000; Yang and Weber, 2019). This is inextricably linked to China’s Confucian culture. Consumers may feel that their individual efforts do not contribute to the improvement of such environmental issues in high power distance cultural contexts (Alzubaidi et al., 2021). However, even within the same cultural context, there are differences in the cultural perceptions of different consumers (Mi et al., 2020), which lead to different pro-environmental behaviors.

### 5.3 Managerial implications

For managerial implications, this study indicates that consumers’ perceptions of COVID-19 risk are associated with a variety of environmental affective responses, which can influence individuals’ attitudes toward the environment and, in turn, their pro-environmental behavior. Consequently, governments and marketers should pay attention to cognitive and emotional factors and their interactions to promote pro-environmental behavior among consumers. In the context of COVID-19, fears and negative messages about ecological problems can be included in marketing to induce consumers’ perception of COVID-19 risks so that consumers consciously attribute the degradation of the ecological environment to human failure in properly handling the moral relationship between humans and nature. In turn, this generates eco-ethical reflections and realizes the relationship between their own abilities and obligations, triggering consumers’ feelings of empathy and guilt. Furthermore, public service announcements and green advertisements on social media may be infused with awe or nostalgia. Inducing transcendent emotions such as awe and nostalgia can increase public awareness of the detrimental consequences of environmentally harmful behavior and attribute responsibility to them, as well as change consumer attitudes toward environmental protection, thereby contributing to pro-environmental behavior.

In countries with higher power distances, consumers are often less enthusiastic about ecological issues, believing that such costly public goods with no short-term benefits should be handled by the government or large corporations. In light of the findings of this study, Chinese consumers should develop a sense of environmental responsibility. Individuals who display environmental responsibility are more likely to take action to mitigate environmental problems. It is a result of their awareness of environmental problems and their perception of the importance of protecting the environment. The government or marketers should appeal to consumers’ concern for the environment in a higher power distance cultural context so that they will be motivated to express an emotional response, which, in turn, enhances their sense of environmental responsibility and drives pro-environmental behavior.

### 5.4 Limitations and future research directions

First, the mechanism through which COVID-19 influences consumers’ pro-environmental behavior is relatively complex. The purpose of this study is to construct a theoretical model based on a cognitive-emotional-attitudinal-behavioral model and affective event theory to examine the psychological mechanisms underlying consumers’ pro-environmental behavior development. Future research can be conducted to comprehensively investigate the mechanism of COVID-19’s impact on consumers’ pro-environmental behavior by combining cognitive and emotional frameworks and different theoretical perspectives.

Second, this paper uses the questionnaire method, and the data are all from self-reports of the same subjects. Although the results of the data analysis indicate that CMB does not pose a significant threat, the rigor of the methodology could be further improved (Sharma et al., 2022a). It is possible for future research on consumer pro-environmental behavior to be supplemented by objective and subjective data to test the robustness of the findings.

To verify the model, this study concludes with a comprehensive application of SEM and a necessity analysis. Researchers may be able to further explore and expand the research model of consumers’ pro-environmental behavior by employing experimental methods and case studies in the future. Furthermore, the majority of research on consumer pro-environmental behavior is based on the analysis of correlations between influencing factors; COVID-19 is in the midst of a natural experiment (Severo et al., 2021b); therefore, future research can use causal inference methods to explore the mechanisms that influence consumers’ pro-environmental behavior.

## Data availability statement

The raw data supporting the conclusions of this article will be made available by the authors, without undue reservation.

## Author contributions

SZ: conceptualization, methodology, data curation, writing—original draft, figures production, and writing—review and editing.

## References

[B1] Adams I. Hurst K. Sintov N. D. (2020). Experienced guilt, but not pride, mediates the effect of feedback on pro-environmental behavior. *J. Environ. Psychol.* 71:101476.

[B2] Ágoston C. Urbán R. Nagy B. Csaba B. Kõváry Z. Kovács K. (2022). The psychological consequences of the ecological crisis: Three new questionnaires to assess eco-anxiety, eco-guilt, and ecological grief. *Clim. Risk Manag.* 37:100441. 10.1016/j.crm.2022.100441

[B3] Ajzen I. (1991). The theory of planned behavior. *Organ. Behav. Hum. Decis. Process.* 50 179–211. 10.1016/0749-5978(91)90020-T

[B4] Alzubaidi H. Slade E. L. Dwivedi Y. K. (2021). Examining antecedents of consumers’ pro-environmental behaviours: TPB extended with materialism and innovativeness. *J. Bus. Res.* 122 685–699. 10.1016/j.jbusres.2020.01.017

[B5] Bagozzi R. P. Yi Y. Phillips L. W. (1991). Assessing construct validity in organizational research. *Adm. Sci. Q.* 36 421–458. 10.2307/2393203

[B6] Bamberg S. Möser G. (2007). Twenty years after Hines, Hungerford, and Tomera: A new meta-analysis of psycho-social determinants of pro-environmental behaviour. *J. Environ. Psychol.* 27 14–25. 10.1016/j.jenvp.2006.12.002

[B7] Boym S. (2008). *The Future of Nostalgia.* New York, NY: Basic books.

[B8] Büssing A. Rodrigues Recchia D. Dienberg T. Surzykiewicz J. Baumann K. (2021). Awe/Gratitude as an Experiential Aspect of Spirituality and Its Association to Perceived Positive Changes During the COVID-19 Pandemic. *Front. Psychiatry* 12:642716. 10.3389/fpsyt.2021.642716 33959049 PMC8095710

[B9] Carrión G. C. Nitzl C. Roldán J. L. (2017). “Mediation Analyses in Partial Least Squares Structural Equation Modeling: Guidelines and Empirical Examples,” in *Partial Least Squares Path Modeling*, eds Latan H. Noonan R. (Cham: Springer International Publishing), 173–195.

[B10] Casaló L. V. Escario J.-J. Rodriguez-Sanchez C. (2019). Analyzing differences between different types of pro-environmental behaviors: Do attitude intensity and type of knowledge matter? *Resour. Conserv. Recycle.* 149 56–64. 10.1016/j.resconrec.2019.05.024

[B11] Cave J. Dredge D. (2020). Regenerative tourism needs diverse economic practices. *Tour. Geogr.* 22 503–513. 10.1080/14616688.2020.1768434

[B12] Chan R. Y. K. (2000). An Emerging Green Market in China: Myth or Reality? *Bus. Horiz.* 43 55–60.

[B13] Chen M. F. (2020b). Moral extension of the protection motivation theory model to predict climate change mitigation behavioral intentions in Taiwan. *Environ. Sci. Pollut. Res. Int.* 27 13714–13725. 10.1007/s11356-020-07963-6 32034589

[B14] Chen M.-F. (2020a). Effects of psychological distance perception and psychological factors on pro-environmental behaviors in Taiwan: Application of construal level theory. *Int. Sociol.* 35 70–89. 10.1177/0268580919881870

[B15] Chen S. Wan F. Yang S. (2022). Normative misperceptions regarding pro-environmental behavior: Mediating roles of outcome efficacy and problem awareness. *J. Environ. Psychol.* 84:101917. 10.1016/j.jenvp.2022.101917

[B16] Chen S. Yang J. Yang W. Wang C. Bärnighausen T. (2020). COVID-19 control in China during mass population movements at New Year. *Lancet* 395 764–766. 10.1016/S0140-6736(20)30421-9 32105609 PMC7159085

[B17] Cheung M. F. Y. To W. M. (2019). An extended model of value-attitude-behavior to explain Chinese consumers’ green purchase behavior. *J. Retail. Consum. Serv.* 50 145–153. 10.1016/j.jretconser.2019.04.006

[B18] Chi N. T. K. (2021). Innovation capability: The impact of e-CRM and COVID-19 risk perception. *Technol. Soc.* 67:101725. 10.1016/j.techsoc.2021.101725

[B19] Chin W. W. Marcolin B. L. Newsted P. R. (2003). A Partial Least Squares Latent Variable Modeling Approach for Measuring Interaction Effects: Results from a Monte Carlo Simulation Study and an Electronic-Mail Emotion/Adoption Study. *Inf. Syst. Res.* 14 189–217. 10.1287/isre.14.2.189.16018 19642375

[B20] Christou P. Farmaki A. Evangelou G. (2018). Nurturing nostalgia?: A response from rural tourism stakeholders. *Tour. Manag.* 69 42–51. 10.1016/j.tourman.2018.05.010

[B21] Cohen J. (1988). *Edition 2. Statistical Power Analysis for the Behavioral Sciences.* Hillsdale: Erlbaum.

[B22] Corraliza J. A. Berenguer J. (2000). Environmental values, beliefs, and actions: A situational approach. *Environ. Behav.* 32 832–848. 10.1097/00012272-200304000-00003 12795539

[B23] Daryanto A. Song Z. Soopramanien D. (2022). The COVID-19 pandemic as an impetus for pro-environmental behaviours: The role of causal attribution. *Pers. Individual Differ.* 187:111415. 10.1016/j.paid.2021.111415 34876764 PMC8639201

[B24] De Silva M. Wang P. Kuah A. T. H. (2021). Why wouldn’t green appeal drive purchase intention? Moderation effects of consumption values in the UK and China. *J. Bus. Res.* 122 713–724. 10.1016/j.jbusres.2020.01.016

[B25] Dendler L. Dewick P. (2016). Institutionalising the organic labelling scheme in China: A legitimacy perspective. *J. Clean. Prod.* 134 239–250. 10.1016/j.jclepro.2016.02.141

[B26] Ducoffe R. H. (1995). How Consumers Assess the Value of Advertising. *J. Curr. Issues Res. Advert.* 17 1–18. 10.1080/10641734.1995.10505022

[B27] Dul J. (2016). Necessary condition analysis (NCA) logic and methodology of “necessary but not sufficient” causality. *Organ. Res. Methods* 19 10–52. 10.1177/1094428115584005

[B28] Dul J. Van der Laan E. Kuik R. (2020). A statistical significance test for necessary condition analysis. *Organ. Res. Methods* 23 385–395. 10.1177/1094428118795272

[B29] Dul J. Vis B. Goertz G. (2018). Necessary Condition Analysis (NCA) Does Exactly What It Should Do When Applied Properly: A Reply to a Comment on NCA*. *Sociol. Methods Res.* 50 926–936. 10.1177/0049124118799383

[B30] Eisenberg N. Cumberland A. Guthrie I. K. Murphy B. C. Shepard S. A. (2005). Age Changes in Prosocial Responding and Moral Reasoning in Adolescence and Early Adulthood. *J. Res. Adolesc.* 15 235–260. 10.1111/j.1532-7795.2005.00095.x 20592955 PMC2893741

[B31] Eom K. Tok T. Q. H. Saad C. S. Kim H. S. (2021). Religion, environmental guilt, and pro-environmental support: The opposing pathways of stewardship belief and belief in a controlling god. *J. Environ. Psychol.* 78:101717. 10.1016/j.jenvp.2021.101717

[B32] Escadas M. Jalali M. S. Farhangmehr M. (2019). Why bad feelings predict good behaviours: The role of positive and negative anticipated emotions on consumer ethical decision making. *Bus. Ethics* 28 529–545.

[B33] Felix R. González E. M. Castaño R. Carrete L. Gretz Richard T. (2022). When the green in green packaging backfires: Gender effects and perceived masculinity of environmentally friendly products. *Int. J. Consum. Stud.* 46 925–943. 10.1111/ijcs.12738

[B34] Festinger L. (1962). *A Theory of Cognitive Dissonance.* California: Stanford university press.

[B35] Fornell C. Larcker D. F. (1981). *Structural Equation Models with Unobservable Variables and Measurement Error: Algebra and Statistics.* Los Angeles, CA: Sage Publications.

[B36] Gezhi C. Xiang H. (2022). From good feelings to good behavior: Exploring the impacts of positive emotions on tourist environmentally responsible behavior. *J. Hosp. Tour. Manag.* 50 1–9. 10.1016/j.jhtm.2021.11.017

[B37] Gibbs H. Egermann H. (2021). Music-Evoked Nostalgia and Wellbeing During the United Kingdom COVID-19 Pandemic: Content, Subjective Effects, and Function. *Front. Psychol.* 12:647891. 10.3389/fpsyg.2021.647891 33828512 PMC8019926

[B38] Gifford R. Nilsson A. (2014). Personal and social factors that influence pro-environmental concern and behaviour: A review. *Int. J. Psychol.* 49 141–157. 10.1002/ijop.12034 24821503

[B39] Gilg A. Barr S. Ford N. (2005). Green consumption or sustainable lifestyles? Identifying the sustainable consumer. *Futures* 37 481–504. 10.1016/j.futures.2004.10.016

[B40] Gu D. Gao S. Wang R. Jiang J. Xu Y. (2020). The negative associations between materialism and pro-environmental attitudes and behaviors: Individual and regional evidence from China. *Environ. Behav.* 52 611–638. 10.1177/0013916518811902

[B41] Gupta S. Gallear D. Rudd J. Foroudi P. (2020). The impact of brand value on brand competitiveness. *J. Bus. Res.* 112 210–222. 10.1016/j.jbusres.2020.02.033

[B42] Haddad M. A. (2015). Increasing environmental performance in a context of low governmental enforcement: Evidence from China. *J. Environ. Dev.* 24 3–25. 10.1177/1070496514564563

[B43] Hair J. F. Jr. Hult G. T. M. Ringle C. M. Sarstedt M. (2021). *A Primer on Partial Least Squares Structural Equation Modeling (PLS-SEM).* Thousand Oaks: Sage publications.

[B44] Hair J. F. Ringle C. M. Sarstedt M. (2013). Partial Least Squares Structural Equation Modeling: Rigorous Applications, Better Results and Higher Acceptance. *Long Range Plan.* 46 1–12. 10.1016/j.lrp.2013.01.001

[B45] Hair J. F. Ringle C. M. Sarstedt M. (2014). PLS-SEM: Indeed a Silver Bullet. *J. Mark. Theory Practice* 19 139–152. 10.2753/mtp1069-6679190202

[B46] Hair J. F. Risher J. J. Sarstedt M. Ringle C. M. (2019). When to use and how to report the results of PLS-SEM. *Eur. Bus. Rev.* 31 2–24. 10.1108/ebr-11-2018-0203

[B47] Haj-Salem N. Ishaq M. I. Raza A. (2022). How anticipated pride and guilt influence green consumption in the Middle East: The moderating role of environmental consciousness. *J. Retail. Consum. Serv.* 68:103062. 10.1016/j.jretconser.2022.103062

[B48] He L. Filimonau V. (2020). The effect of national culture on pro-environmental behavioural intentions of tourists in the UK and China. *Tour. Manag. Perspect.* 35:100716.

[B49] Henseler J. Fassott G. (2010). “Testing Moderating Effects in PLS Path Models: An Illustration of Available Procedures,” in *Handbook of Partial Least Squares*, eds Esposito Vinzi V. Chin W. W. Henseler J. Wang H. (Berlin: Springer), 713–735.

[B50] Henseler J. Hubona G. Ray P. A. (2016). Using PLS path modeling in new technology research: Updated guidelines. *Ind. Manag. Data Syst.* 116 2–20. 10.1108/imds-09-2015-0382

[B51] Henseler J. Ringle C. M. Sarstedt M. (2014). A new criterion for assessing discriminant validity in variance-based structural equation modeling. *J. Acad. Mark. Sci.* 43 115–135. 10.1007/s11747-014-0403-8

[B52] Henseler J. Ringle C. M. Sarstedt M. (2015). A new criterion for assessing discriminant validity in variance-based structural equation modeling. *J. Acad. Mark. Sci.* 43 115–135.

[B53] Herbes C. Beuthner C. Ramme I. (2020). How green is your packaging—A comparative international study of cues consumers use to recognize environmentally friendly packaging. *Int. J. Consum. Stud.* 44 258–271. 10.1111/ijcs.12560

[B54] Hofstede G. (1984). Cultural dimensions in management and planning. *Asia Pac. J. Manag.* 1 81–99. 10.1007/bf01733682

[B55] Hofstede G. (1989). Organising for cultural diversity. *Eur. Manag. J.* 7 390–397. 10.1016/0263-2373(89)90075-3

[B56] Hua Y. Dong F. (2022). Can environmental responsibility bridge the intention-behavior gap? Conditional process model based on valence theory and the theory of planned behavior. *J. Clean. Prod.* 376:134166.

[B57] Huang L. Guo Z. Deng B. Wang B. (2022). Unlocking the relationship between environmentally specific transformational leadership and employees’ green behaviour: A cultural self-representation perspective. *J. Clean. Prod.* 382:134857. 10.1016/j.jclepro.2022.134857

[B58] Ienna M. Rofe A. Gendi M. Douglas H. E. Kelly M. Hayward M. W. (2022). The Relative Role of Knowledge and Empathy in Predicting Pro-Environmental Attitudes and Behavior. *Sustainability* 14:4622. 10.3390/su14084622

[B59] Im J. Kim J. Choeh J. Y. (2021). COVID-19, social distancing, and risk-averse actions of hospitality and tourism consumers: A case of South Korea. *J. Dest. Mark. Manage.* 20:100566. 10.1016/j.jdmm.2021.100566

[B60] Jacobs T. P. McConnell A. R. (2022). Self-transcendent emotion dispositions: Greater connections with nature and more sustainable behavior. *J. Environ. Psychol.* 81:101797. 10.1016/j.jenvp.2022.101797

[B61] Kaiser F. G. (2021). Climate change mitigation within the Campbell paradigm: Doing the right thing for a reason and against all odds. *Curr. Opin. Behav. Sci.* 42 70–75.

[B62] Kapoor K. K. Dwivedi Y. K. (2020). Sustainable consumption from the consumer’s perspective: Antecedents of solar innovation adoption. *Resour. Conserv. Recycle.* 152:104501. 10.1016/j.resconrec.2019.104501

[B63] Kaynak E. Wong Y. Leung T. (2013). *Guanxi: Relationship Marketing in a Chinese Context.* Milton Park: Routledge.

[B64] Keltner D. Haidt J. (2003). Approaching awe, a moral, spiritual, and aesthetic emotion. *Cogn. Emot.* 17 297–314. 10.1080/02699930302297 29715721

[B65] Kim J. Yang K. Min J. White B. (2022). Hope, fear, and consumer behavioral change amid COVID-19: Application of protection motivation theory. *Int. J. Consum. Stud.* 46 558–574. 10.1111/ijcs.12700 34220343 PMC8237022

[B66] Kirkman B. L. Chen G. Farh J.-L. Chen Z. X. Lowe K. B. (2009). Individual power distance orientation and follower reactions to transformational leaders: A cross-level, cross-cultural examination. *Acad. Manag. J.* 52 744–764.

[B67] Koenig-Lewis N. Palmer A. Dermody J. Urbye A. (2014). Consumers’ evaluations of ecological packaging–Rational and emotional approaches. *J. Environ. Psychol.* 37 94–105.

[B68] Kollmuss A. Agyeman J. (2002). Mind the gap: Why do people act environmentally and what are the barriers to pro-environmental behavior? *Environ. Educ. Res.* 8 239–260. 10.1080/13504620220145401

[B69] Kwon S. Park A. (2022). Understanding user responses to the COVID-19 pandemic on Twitter from a terror management theory perspective: Cultural differences among the US, UK and India. *Comput. Hum. Behav.* 128:107087. 10.1016/j.chb.2021.107087 34744298 PMC8558263

[B70] Lawrance E. L. Jennings N. Kioupi V. Thompson R. Diffey J. Vercammen A. (2022). Psychological responses, mental health, and sense of agency for the dual challenges of climate change and the COVID-19 pandemic in young people in the UK: An online survey study. *Lancet Planet. Health* 6 e726–e738. 10.1016/S2542-5196(22)00172-3 36087603 PMC9451498

[B71] Leong L.-Y. Hew T.-S. Ooi K.-B. Dwivedi Y. K. (2020b). Predicting trust in online advertising with an SEM-artificial neural network approach. *Expert Syst. Appl.* 162:113849.

[B72] Leong L.-Y. Hew T.-S. Ooi K.-B. Chong A. Y.-L. (2020a). Predicting the antecedents of trust in social commerce – A hybrid structural equation modeling with neural network approach. *J. Bus. Res.* 110 24–40. 10.1016/j.jbusres.2019.11.056

[B73] Li J. Hallsworth A. G. Coca-Stefaniak J. A. (2020). Changing grocery shopping behaviours among Chinese consumers at the outset of the COVID-19 outbreak. *J. Econ. Hum. Geography* 111 574–583. 10.1111/tesg.12420 32836486 PMC7307130

[B74] Li J. Zhang J. Zhang D. Ji Q. (2019). Does gender inequality affect household green consumption behaviour in China? *Energy Policy* 135:111071.

[B75] Lim J. Moon K.-K. (2022). Does Political Participation Strengthen the Relationship between Civic Morality and Environmentally Friendly Attitudes? Evidence from South Korea. *Int. J. Environ. Res. Public Health* 19:2095. 10.3390/ijerph19042095 35206283 PMC8872144

[B76] Lin M.-T. Zhu D. Liu C. Kim P. B. (2022). A meta-analysis of antecedents of pro-environmental behavioral intention of tourists and hospitality consumers. *Tour. Manag.* 93:104566. 10.1016/j.tourman.2022.104566

[B77] Linder N. Rosenthal S. Sorqvist P. Barthel S. (2021). Internal and External Factors’. Influence on Recycling: Insights From a Laboratory Experiment With Observed Behavior. *Front. Psychol.* 12:699410. 10.3389/fpsyg.2021.699410 34367024 PMC8340013

[B78] Liu X. Wen J. Kozak M. Jiang Y. Li Z. (2021). Negotiating interdisciplinary practice under the COVID-19 crisis: Opportunities and challenges for tourism research. *Tour. Rev.* 77 484–502.

[B79] Lucarelli C. Mazzoli C. Severini S. (2020). Applying the theory of planned behavior to examine pro-environmental behavior: The moderating effect of COVID-19 beliefs. *Sustainability* 12:10556. 10.3390/su122410556

[B80] Luo M. M. Chea S. (2018). Cognitive appraisal of incident handling, affects, and post-adoption behaviors: A test of affective events theory. *Int. J. Inf. Manage.* 40 120–131. 10.1016/j.ijinfomgt.2018.01.014

[B81] Maleksaeidi H. Keshavarz M. (2019). What influences farmers’ intentions to conserve on-farm biodiversity? An application of the theory of planned behavior in fars province, Iran. *Glob. Ecol. Conserv.* 20:e00698. 10.1016/j.gecco.2019.e00698

[B82] Meyer A. (2015). Does education increase pro-environmental behavior? *Evidence Eur. Ecol. Econ.* 116 108–121. 10.1016/j.ecolecon.2015.04.018

[B83] Mi L. Qiao L. Xu T. Gan X. Yang H. Zhao J. (2020). Promoting sustainable development: The impact of differences in cultural values on residents’ pro-environmental behaviors. *Sustain. Dev.* 28 1539–1553. 10.1002/sd.2103

[B84] Mi L. Zhao J. Xu T. Yang H. Lv T. Shang K. (2021). How does COVID-19 emergency cognition influence public pro-environmental behavioral intentions? An affective event perspective. *Resour. Conserv. Recycle.* 168 105467. 10.1016/j.resconrec.2021.105467 33564208 PMC7857117

[B85] Milfont T. L. Osborne D. Sibley C. G. (2022a). Socio-political efficacy explains increase in New Zealanders’ pro-environmental attitudes due to COVID-19. *J. Environ. Psychol.* 79:101751. 10.1016/j.jenvp.2021.101751 35002011 PMC8720917

[B86] Milfont T. L. Osborne D. Sibley C. G. (2022b). Socio-political efficacy explains increase in New Zealanders’ pro-environmental attitudes due to COVID-19. *J. Environ. Psychol.* 79:101751. 10.1016/j.jenvp.2021.101751 PMC8720917 35002011

[B87] Moon M. A. Mohel S. H. Farooq A. (2021). I green, you green, we all green: Testing the extended environmental theory of planned behavior among the university students of Pakistan. *J. Soc. Sci.* 58 316–332. 10.1016/j.soscij.2019.05.001

[B88] Newman G. E. Bloom P. Knobe J. (2014). Value Judgments and the True Self. *Pers. Soc. Psychol. Bull.* 40 203–216. 10.1177/0146167213508791 24154918

[B89] Nielsen K. S. Brick C. Hofmann W. Joanes T. Lange F. Gwozdz W. (2022). The motivation–impact gap in pro-environmental clothing consumption. *Nat. Sustain.* 5 665–668. 10.1038/s41893-022-00888-7

[B90] O’Connor P. Assaker G. (2022). COVID-19’s effects on future pro-environmental traveler behavior: An empirical examination using norm activation, economic sacrifices, and risk perception theories. *J. Sustain. Tour.* 30 89–107. 10.1080/09669582.2021.1879821

[B91] Onwezen M. C. Bartels J. Antonides G. (2014). Environmentally friendly consumer choices: Cultural differences in the self-regulatory function of anticipated pride and guilt. *J. Environ. Psychol.* 40 239–248. 10.1016/j.jenvp.2014.07.003

[B92] Pearce J. Huang S. Dowling R. K. Smith A. J. (2022). Effects of social and personal norms, and connectedness to nature, on pro-environmental behavior: A study of Western Australian protected area visitors. *Tour. Manag. Perspect.* 42:100966. 10.1016/j.tmp.2022.100966

[B93] Piff P. K. Dietze P. Feinberg M. Stancato D. M. Keltner D. (2015). Awe, the small self, and prosocial behavior. *J. Pers. Soc. Psychol.* 108 883–899. 10.1037/pspi0000018 25984788

[B94] Podsakoff P. M. MacKenzie S. B. Lee J. Y. Podsakoff N. P. (2003). Common method biases in behavioral research: A critical review of the literature and recommended remedies. *J. Appl. Psychol.* 88 879–903. 10.1037/0021-9010.88.5.879 14516251

[B95] Polonsky M. J. Vocino A. Grau S. L. Garma R. Ferdous A. S. (2012). The impact of general and carbon-related environmental knowledge on attitudes and behaviour of US consumers. *J. Mark. Manag.* 28 238–263. 10.1080/0267257X.2012.659279

[B96] Poortinga W. Steg L. Vlek C. (2004). Values, environmental concern, and environmental behavior: A study into household energy use. *Environ. Behav.* 36 70–93. 10.1177/0013916518807963

[B97] Prade C. Saroglou V. (2016). Awe’s effects on generosity and helping. *J. Posit. Psychol.* 11 522–530. 10.1080/17439760.2015.1127992

[B98] Price J. C. Walker I. A. Boschetti F. (2014). Measuring cultural values and beliefs about environment to identify their role in climate change responses. *J. Environ. Psychol.* 37 8–20. 10.1016/j.jenvp.2013.10.001

[B99] Punzo G. Panarello D. Pagliuca M. M. Castellano R. Aprile M. C. (2019). Assessing the role of perceived values and felt responsibility on pro-environmental behaviours: A comparison across four EU countries. *Environ. Sci. Policy* 101 311–322. 10.1016/j.envsci.2019.09.006

[B100] Qin Q. Hsu C. H. C. (2022). Urban travelers’ pro-environmental behaviors: Composition and role of pro-environmental contextual force. *Tour. Manag.* 92:104561. 10.1016/j.tourman.2022.104561

[B101] Ramkissoon H. (2020). COVID-19 Place Confinement, Pro-Social, Pro-environmental Behaviors, and Residents’ Wellbeing: A New Conceptual Framework. *Front. Psychol.* 11:2248. 10.3389/fpsyg.2020.02248 32982895 PMC7490327

[B102] Ramus C. A. Killmer A. B. (2007). Corporate greening through prosocial extrarole behaviours–a conceptual framework for employee motivation. *Bus. Strategy Environ.* 16 554–570.

[B103] Rees J. H. Klug S. Bamberg S. (2015). Guilty conscience: Motivating pro-environmental behavior by inducing negative moral emotions. *Clim. Change* 130 439–452. 10.1007/s10584-014-1278-x

[B104] Richter N. F. Schubring S. Hauff S. Ringle C. M. Sarstedt M. (2020). When predictors of outcomes are necessary: Guidelines for the combined use of PLS-SEM and NCA. *Ind. Manag. Data Sys.* 120 2243–2267. 10.1108/IMDS-11-2019-0638

[B105] Rosenmann A. Reese G. Cameron J. E. (2016). Social Identities in a Globalized World: Challenges and Opportunities for Collective Action. *Perspect. Psychol. Sci.* 11 202–221. 10.1177/1745691615621272 26993275

[B106] Rudd M. Vohs K. D. Aaker J. (2012). Awe expands people’s perception of time, alters decision making, and enhances well-being. *Psychol. Sci.* 23 1130–1136. 10.1177/0956797612438731 22886132

[B107] Santos S. Gonçalves H. M. (2019). Multichannel consumer behaviors in the mobile environment: Using fsQCA and discriminant analysis to understand webrooming motivations. *J. Bus. Res.* 101 757–766. 10.1016/j.jbusres.2018.12.069

[B108] Schultz P. W. (2001). The structure of environmental concern: Concern for self, other people, and the biosphere. *J. Environ. Psychol.* 21 327–339. 10.3389/fpsyg.2019.00453 30899233 PMC6416211

[B109] Schwartz D. Loewenstein G. Agüero-Gaete L. (2020). Encouraging pro-environmental behaviour through green identity labelling. *Nat. Sustain.* 3 746–752. 10.1038/s41893-020-0543-4

[B110] Sedikides C. Wildschut T. Arndt J. Routledge C. (2008). Nostalgia: Past. Present, and Future. *Curr. Dir. Psychol. Sci.* 17 304–307. 10.1111/j.1467-8721.2008.00595.x

[B111] Severo E. A. De Guimaraes J. C. F. Dellarmelin M. L. (2021a). Impact of the COVID-19 pandemic on environmental awareness, sustainable consumption and social responsibility: Evidence from generations in Brazil and Portugal. *J. Clean. Prod.* 286:124947. 10.1016/j.jclepro.2020.124947 33173257 PMC7644235

[B112] Severo E. A. De Guimarães J. C. F. Dellarmelin M. L. (2021b). Impact of the COVID-19 pandemic on environmental awareness, sustainable consumption and social responsibility: Evidence from generations in Brazil and Portugal. *J. Clean. Prod.* 286:124947. 10.1016/j.jclepro.2020.124947 PMC7644235 33173257

[B113] Shakil M. H. Munim Z. H. Tasnia M. Sarowar S. (2020). COVID-19 and the environment: A critical review and research agenda. *Sci. Total Environ.* 745:141022. 10.1016/j.scitotenv.2020.141022 32711074 PMC7366970

[B114] Sharma N. Lal M. Goel P. Sharma A. Rana N. P. (2022b). Being socially responsible: How green self-identity and locus of control impact green purchasing intentions? *J. Clean. Prod.* 357:131895. 10.1016/j.jclepro.2022.131895

[B115] Sharma A. Dwivedi R. Mariani M. M. Islam T. (2022a). Investigating the effect of advertising irritation on digital advertising effectiveness: A moderated mediation model. *Technol. Forecast. Soc. Change* 180:121731. 10.1016/j.techfore.2022.121731

[B116] Shi H. Wang S. Guo S. (2019). Predicting the impacts of psychological factors and policy factors on individual’s PM2.5 reduction behavior: An empirical study in China. *J. Clean. Prod.* 241:118416. 10.1016/j.jclepro.2019.118416

[B117] Shiota M. N. Keltner D. John O. P. (2006). Positive emotion dispositions differentially associated with Big Five personality and attachment style. *J. Posit. Psychol.* 1 61–71. 10.1080/17439760500510833

[B118] Shipley N. J. van Riper C. J. (2021). Pride and guilt predict pro-environmental behavior: A meta-analysis of correlational and experimental evidence. *J. Environ. Psychol.* 79:101753.

[B119] Shulman D. Halperin E. Reifen-Tagar M. (2022). Personal experience with Covid-19 is associated with increased environmental concern and pro-environmental behavioral intentions. *Curr. Res. Ecol. Soc. Psychol.* 3:100031. 10.1016/j.cresp.2021.100031 35098190 PMC8720047

[B120] Simpson B. Maguire M. Schermer J. A. (2021). Predicting pro-environmental values and behaviors with the supernumerary personality inventory and hope. *Pers. Individual Differ.* 181:111051. 10.1016/j.paid.2021.111051 PMC7427592 32834291

[B121] Spiller S. A. Fitzsimons G. J. Lynch J. G. Mcclelland G. H. (2013). Spotlights, Floodlights, and the Magic Number Zero: Simple Effects Tests in Moderated Regression. *J. Mark. Res.* 50 277–288. 10.1509/jmr.12.0420 11670861

[B122] Sreen N. Purbey S. Sadarangani P. (2018). Impact of culture, behavior and gender on green purchase intention. *J. Retail. Consum. Serv.* 41 177–189.

[B123] Srivastava E. Sivakumaran B. Maheswarappa S. S. Paul J. (2022). Nostalgia: A Review, Propositions, and Future Research Agenda. *J. Advert.* 1–20. 10.1080/00913367.2022.2101036

[B124] Stellar J. E. Gordon A. M. Piff P. K. Cordaro D. Anderson C. L. Bai Y. (2017). Self-transcendent emotions and their social functions: Compassion, gratitude, and awe bind us to others through prosociality. *Emot. Rev.* 9 200–207.

[B125] Stern P. C. (2000). New Environmental Theories: Toward a Coherent Theory of Environmentally Significant Behavior. *J. Soc. Issues* 56 407–424. 10.1111/0022-4537.00175

[B126] Sun X. Su W. Guo X. Tian Z. (2021a). The Impact of Awe Induced by COVID-19 Pandemic on Green Consumption Behavior in China. *Int. J. Environ. Res. Public Health* 18:543. 10.3390/ijerph18020543 33440719 PMC7826881

[B127] Sun X. Su W. Guo X. Tian Z. (2021b). The Impact of Awe Induced by COVID-19 Pandemic on Green Consumption Behavior in China. *Int. J. Environ. Res. Public Health* 18:543. 10.3390/ijerph18020543 PMC7826881 33440719

[B128] Sun Y. Leng K. Xiong H. (2022). Research on the influencing factors of consumers’ green purchase behavior in the post-pandemic era. *J. Retail. Consum. Serv.* 69:103118. 10.1016/j.jretconser.2022.103118

[B129] Sun Y. Li P. She S. Eimontaite I. Yang B. (2018). Boosting water conservation by improving campaign: Evidence from a field study in China. *Urban Water J.* 15 966–973.

[B130] Sun Y. Liu N. Zhao M. (2019). Factors and mechanisms affecting green consumption in China: A multilevel analysis. *J. Clean. Prod.* 209 481–493. 10.1016/j.jclepro.2018.10.241

[B131] Tam K.-P. (2013). Concepts and measures related to connection to nature: Similarities and differences. *J. Environ. Psychol.* 34 64–78. 10.1016/j.jenvp.2013.01.004

[B132] Tanner C. (1999). Constraints on environmental behaviour. *J. Environ. Psychol.* 19 145–157. 10.1006/jevp.1999.0121

[B133] Tchetchik A. Kaplan S. Blass V. (2021). Recycling and consumption reduction following the COVID-19 lockdown: The effect of threat and coping appraisal, past behavior and information. *Resour. Conserv. Recycle.* 167:105370. 10.1016/j.resconrec.2020.105370 PMC9759599 36570977

[B134] Teper R. Zhong C. B. Inzlicht M. (2015). How emotions shape moral behavior: Some answers (and questions) for the field of moral psychology. *Soc. Personal. Psychol. Compass* 9 1–14. 10.1111/spc3.12200 PMC5621751 28966660

[B135] Thompson C. L. Kuah A. T. Foong R. Ng E. S. (2020). The development of emotional intelligence, self-efficacy, and locus of control in Master of Business Administration students. *Hum. Resour. Dev. Q.* 31 113–131. 10.1002/hrdq.21375

[B136] Tian H. Liu X. (2022). Pro-Environmental Behavior Research: Theoretical Progress and Future Directions. *Int. J. Environ. Res. Public Health* 19:6721. 10.3390/ijerph19116721 35682302 PMC9180624

[B137] Tversky A. Fox C. R. (1995). Weighing risk and uncertainty. *Psychol. Rev.* 102:269. 10.1037/0033-295X.102.2.269

[B138] Urban J. Braun Kohlová M. (2022). The COVID-19 crisis does not diminish environmental motivation: Evidence from two panel studies of decision making and self-reported pro-environmental behavior. *J. Environ. Psychol.* 80:101761. 10.1016/j.jenvp.2022.101761 35075318 PMC8770255

[B139] Urban J. Braun Kohlova M. (2022). The COVID-19 crisis does not diminish environmental motivation: Evidence from two panel studies of decision making and self-reported pro-environmental behavior. *J. Environ. Psychol.* 80:101761. 10.1016/j.jenvp.2022.101761 PMC8770255 35075318

[B140] Vázquez-Martínez U. J. Morales-Mediano J. Leal-Rodríguez A. L. (2021). The impact of the COVID-19 crisis on consumer purchasing motivation and behavior. *Eur. Res. Manag. Bus. Econ.* 27:100166. 10.1016/j.iedeen.2021.100166

[B141] Vis B. Dul J. (2018). Analyzing Relationships of Necessity Not Just in Kind But Also in Degree:Complementing fsQCA With NCA. *Sociol. Methods Res.* 47 872–899. 10.1177/0049124115626179 30443090 PMC6195096

[B142] Wang L. Zhang G. Shi P. Lu X. Song F. (2019). Influence of Awe on Green Consumption: The Mediating Effect of Psychological Ownership. *Front. Psychol.* 10:2484. 10.3389/fpsyg.2019.02484 31780993 PMC6856648

[B143] Wang X. Chao C.-H. (2019). Nostalgia decreases green consumption: The mediating role of past orientation. *BRQ Bus. Res. Q.* 23 270–284. 10.1016/j.brq.2019.03.005

[B144] Wang Y. Tian H. Sarigöllü E. Xu W. (2020). Nostalgia prompts sustainable product disposal. *J. Consum. Behav.* 19 570–580. 10.1002/cb.1832

[B145] Weiss H. M. Cropanzano R. (1996). Affective events theory. *Res. Organ. Behav.* 18 1–74. 10.1037/0021-9010.79.2.272

[B146] Wetzels M. Odekerken-Schröder G. Van Oppen C. (2009). Using PLS path modeling for assessing hierarchical construct models: Guidelines and empirical illustration. *MIS Q.* 33 177–195. 10.2307/20650284

[B147] Wiernik B. M. Ones D. S. Dilchert S. (2013). Age and environmental sustainability: A meta-analysis. *J. Manag. Psychol.* 28 826–856.

[B148] Wildschut T. Sedikides C. Arndt J. Routledge C. (2006). Nostalgia: Content, triggers, functions. *J. Pers. Soc. Psychol.* 91 975–993. 10.1037/0022-3514.91.5.975 17059314

[B149] Wu J. S. Font X. Liu J. (2021). The elusive impact of pro-environmental intention on holiday on pro-environmental behaviour at home. *Tour. Manag.* 85:104283. 10.1016/j.tourman.2021.104283

[B150] Wu Q. Zhang Y. He W. Cui L. (2022). The relationship between adolescents’ materialism and cooperative propensity: The mediating role of greed and the moderating role of awe. *Pers. Individual Differ.* 189:111484. 10.1016/j.paid.2021.111484

[B151] Xia L. Wang J. Santana S. (2021). Nostalgia: Triggers and its role on new product purchase intentions. *J. Bus. Res.* 135 183–194. 10.1016/j.jbusres.2021.06.034

[B152] Xiang D. Jiao G. Sun B. Peng C. Ran Y. (2022). Prosumer-to-customer exchange in the sharing economy: Evidence from the P2P accommodation context. *J. Bus. Res.* 145 426–441. 10.1016/j.jbusres.2022.02.077

[B153] Yadav R. Balaji M. S. Jebarajakirthy C. (2019). How psychological and contextual factors contribute to travelers’ propensity to choose green hotels? *Int. J. Hosp. Manag.* 77 385–395. 10.1016/j.ijhm.2018.08.002

[B154] Yang X. Jiang J. Chen S. C. (2022). Achieving sustainability: Determinants of conscious green purchasing behavior during the COVID-19 pandemic. *Bus. Strategy Environ.* [Preprint]. 10.1002/bse.3245 36249586 PMC9538399

[B155] Yang X. Weber A. (2019). Who can improve the environment—Me or the powerful others? An integrative approach to locus of control and pro-environmental behavior in China. *Resour. Conserv. Recycle.* 146 55–67. 10.1016/j.resconrec.2019.03.005

[B156] Yang Y. Hu J. (2021). Self-diminishing effects of awe on consumer forgiveness in service encounters. *J. Retail. Consum. Serv.* 60:102491. 10.1016/j.jretconser.2021.102491

[B157] Yin C. Ma H. Gong Y. Chen Q. Zhang Y. (2021). Environmental CSR and environmental citizenship behavior: The role of employees’ environmental passion and empathy. *J. Clean. Prod.* 320:128751. 10.1016/j.jclepro.2021.128751

[B158] Yoo B. Donthu N. Lenartowicz T. (2011). Measuring Hofstede’s Five Dimensions of Cultural Values at the Individual Level: Development and Validation of CVSCALE. *J. Int. Consum. Mark.* 23 193–210. 10.1080/08961530.2011.578059

[B159] Zebardast L. Radaei M. (2022). The influence of global crises on reshaping pro-environmental behavior, case study: The COVID-19 pandemic. *Sci. Total Environ.* 811:151436. 10.1016/j.scitotenv.2021.151436 34742989 PMC8596762

[B160] Zhang B. Lai K.-H. Wang B. Wang Z. (2019). From intention to action: How do personal attitudes, facilities accessibility, and government stimulus matter for household waste sorting? *J. Environ. Manage.* 233 447–458. 10.1016/j.jenvman.2018.12.059 30593004

[B161] Zhang X. Gong X. Jiang J. (2021). Dump or recycle? Nostalgia and consumer recycling behavior. *J. Bus. Res.* 132 594–603. 10.1016/j.jbusres.2020.11.033

[B162] Zhang Y. Tao W. (2022). The Impact of Nostalgia Proneness on Online Donation Willingness: The Mediating Effect of Consumer-Brand Relationship. *Front. Psychol.* 13:927330. 10.3389/fpsyg.2022.927330 35769739 PMC9236181

[B163] Zhao H. Zhang H. Xu Y. Lu J. He W. (2018). Relation between awe and environmentalism: The role of social dominance orientation. *Front. Psychol.* 9:2367. 10.3389/fpsyg.2018.02367 30559692 PMC6286991

[B164] Zhong C.-B. Ku G. Lount R. B. Murnighan J. K. (2010). Compensatory ethics. *J. Bus. Ethics* 92 323–339. 10.1007/s10551-009-0161-6

[B165] Zhou H. Yin H. Yuan F. Wang F. (2019). Social relationships, public media, and pro-environmental behaviors. *Empir. Econ.* 57 569–588. 10.1007/s00181-018-1499-3

[B166] Zhou X. Sedikides C. Mo T. Li W. Hong E. K. Wildschut T. (2022). The Restorative Power of Nostalgia: Thwarting Loneliness by Raising Happiness During the COVID-19 Pandemic. *Soc. Psychol. Personal. Sci.* 13 803–815. 10.1177/19485506211041830

