# Supplementary material for: The effect of COVID-19 risk perception on pro-environmental behavior of Chinese consumers: Perspectives from affective event theory
Source: Front Psychol. 2023 Jan 4;13:1093999. doi: 10.3389/fpsyg.2022.1093999 (PMC9845731; doi:10.3389/fpsyg.2022.1093999)
Supplement: Supplementary file 1 [file Data_Sheet_1.docx]

# Appendix:

Appendix 1 Description of research item

Appendix 2 Inner model result (control variable added)

Appendix 3 Screenshot of Inner model result (control variable added)

Appendix 4 Normality test

Appendix 5 QQ plot

Appendix 6 Cross loading result

Appendix 7 Research items

Appendix 8 G Power screen shot

| Appendix 1 description of research item | | | | | |
| --- | --- | --- | --- | --- | --- |
| Name | Mean | Median | Standard deviation | Excess kurtosis | Skewness |
| GU1 | 3.523 | 4 | 1.282 | -0.75 | -0.636 |
| GU2 | 3.301 | 3 | 1.19 | -1.01 | -0.143 |
| GU3 | 3.481 | 4 | 1.249 | -0.848 | -0.512 |
| GU4 | 3.337 | 4 | 1.278 | -0.85 | -0.495 |
| AT1 | 3.647 | 4 | 0.995 | 0.686 | -0.976 |
| AT2 | 3.707 | 4 | 1.139 | -0.054 | -0.822 |
| AT3 | 3.653 | 4 | 1.147 | -0.013 | -0.851 |
| PEB1 | 3.214 | 4 | 1.343 | -1.401 | -0.107 |
| PEB2 | 3.112 | 3 | 1.375 | -1.363 | -0.027 |
| PEB3 | 3.016 | 3 | 1.375 | -1.337 | -0.126 |
| PEB4 | 2.645 | 2 | 1.253 | -0.808 | 0.445 |
| PEB5 | 2.914 | 3 | 1.328 | -1.162 | 0.143 |
| AN1 | 3.196 | 3 | 1.299 | -1.209 | -0.181 |
| AN2 | 3.092 | 3 | 1.312 | -1.214 | -0.09 |
| AN3 | 3.25 | 4 | 1.359 | -1.242 | -0.248 |
| NO1 | 3.643 | 4 | 1.193 | -0.13 | -0.889 |
| NO2 | 3.565 | 4 | 1.181 | -0.559 | -0.607 |
| NO3 | 3.647 | 4 | 1.235 | -0.354 | -0.795 |
| CRP1 | 3.473 | 4 | 1.278 | -0.89 | -0.627 |
| CRP2 | 3.379 | 4 | 1.282 | -1.051 | -0.371 |
| CRP3 | 3.437 | 4 | 1.341 | -1.109 | -0.455 |
| CRP4 | 3.164 | 3 | 1.227 | -0.916 | -0.224 |
| CRP5 | 3.07 | 3 | 1.15 | -0.73 | -0.09 |
| CRP6 | 3.375 | 4 | 1.2 | -0.696 | -0.497 |
| PD1 | 3.008 | 3 | 1.292 | -1.215 | 0.041 |
| PD2 | 2.898 | 3 | 1.265 | -1.105 | 0.216 |
| PD3 | 2.98 | 3 | 1.396 | -1.31 | 0.08 |
| PD4 | 3.068 | 3 | 1.413 | -1.311 | -0.023 |
| PD5 | 3.078 | 3 | 1.453 | -1.357 | -0.073 |

| Appendix 2 Inner model result (control variable added) | | | | |
| --- | --- | --- | --- | --- |
| Path | Original sample (O) | Standard deviation  (STDEV) | T statistics  (\|O/STDEV\|) | P values |
| **Edu -> PEB** | **0.048** | **0.031** | **1.539** | **0.124** |
| **Age -> PEB** | **-0.05** | **0.032** | **1.554** | **0.12** |
| **Gender -> PEB** | **-0.075** | **0.065** | **1.166** | **0.244** |
| **Income -> PEB** | **-0.032** | **0.034** | **0.935** | **0.35** |
| AN -> AT | 0.142 | 0.039 | 3.683 | *** |
| AN -> PEB | 0.176 | 0.042 | 4.148 | *** |
| AT -> PEB | 0.241 | 0.05 | 4.8 | *** |
| CRP -> AN | 0.604 | 0.031 | 19.788 | *** |
| CRP -> GU | 0.631 | 0.031 | 20.06 | *** |
| CRP -> NO | 0.588 | 0.035 | 16.902 | *** |
| CRP -> PEB | 0.137 | 0.047 | 2.885 | 0.004 |
| GU -> AT | 0.125 | 0.044 | 2.865 | 0.004 |
| NO -> AT | 0.521 | 0.042 | 12.328 | *** |
| NO -> PEB | 0.156 | 0.052 | 3.026 | 0.002 |
| PD -> PEB | -0.335 | 0.036 | 9.295 | *** |
| PD x AN -> PEB | 0.049 | 0.039 | 1.254 | 0.21 |
| PD x NO -> PEB | -0.122 | 0.047 | 2.621 | 0.009 |
| PD x CRP -> PEB | 0.006 | 0.044 | 0.125 | 0.901 |
| PD x AT -> PEB | -0.138 | 0.043 | 3.235 | 0.001 |


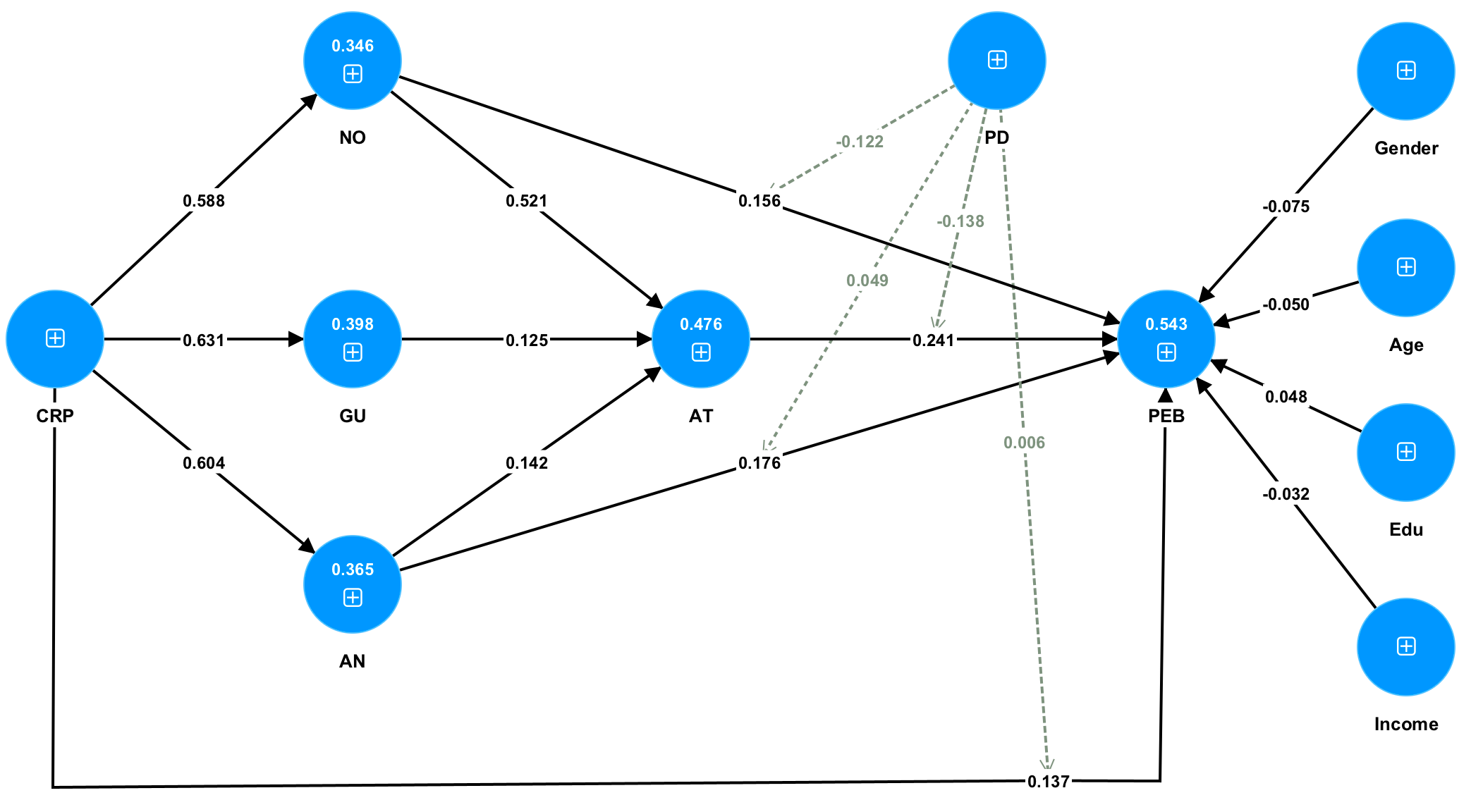


Appendix 3 Control variable added (Screen shot)

Appendix 4: Normality test

| One-Sample Kolmogorov-Smirnov Test | | | | | | | |
| --- | --- | --- | --- | --- | --- | --- | --- |
| Item | N | Normal Parameters | | Most Extreme Differences | | | Asymp. Sig.  (2-tailed) |
|  |  | Mean | Std. Deviation | Absolute | Positive | Negative |  |
| GU1 | 501 | 3.52 | 1.283 | 0.278 | 0.136 | -0.278 | 0.000 |
| GU2 | 501 | 3.3 | 1.191 | 0.19 | 0.158 | -0.19 | 0.000 |
| GU3 | 501 | 3.48 | 1.25 | 0.256 | 0.143 | -0.256 | 0.000 |
| GU4 | 501 | 3.34 | 1.279 | 0.249 | 0.121 | -0.249 | 0.000 |
| AT1 | 501 | 3.65 | 0.996 | 0.321 | 0.214 | -0.321 | 0.000 |
| AT2 | 501 | 3.71 | 1.14 | 0.272 | 0.139 | -0.272 | 0.000 |
| AT3 | 501 | 3.65 | 1.149 | 0.287 | 0.154 | -0.287 | 0.000 |
| PEB1 | 501 | 3.21 | 1.345 | 0.24 | 0.238 | -0.24 | 0.000 |
| PEB2 | 501 | 3.11 | 1.377 | 0.215 | 0.215 | -0.202 | 0.000 |
| PEB3 | 501 | 3.02 | 1.377 | 0.232 | 0.181 | -0.232 | 0.000 |
| PEB4 | 501 | 2.64 | 1.254 | 0.223 | 0.223 | -0.114 | 0.000 |
| PEB5 | 501 | 2.91 | 1.329 | 0.191 | 0.191 | -0.15 | 0.000 |
| AN1 | 501 | 3.2 | 1.3 | 0.227 | 0.188 | -0.227 | 0.000 |
| AN2 | 501 | 3.09 | 1.313 | 0.205 | 0.184 | -0.205 | 0.000 |
| AN3 | 501 | 3.25 | 1.361 | 0.224 | 0.174 | -0.224 | 0.000 |
| NO1 | 501 | 3.64 | 1.194 | 0.312 | 0.153 | -0.312 | 0.000 |
| NO2 | 501 | 3.56 | 1.182 | 0.258 | 0.127 | -0.258 | 0.000 |
| NO3 | 501 | 3.65 | 1.237 | 0.275 | 0.137 | -0.275 | 0.000 |
| CRP1 | 501 | 3.47 | 1.28 | 0.328 | 0.179 | -0.328 | 0.000 |
| CRP2 | 501 | 3.38 | 1.284 | 0.239 | 0.16 | -0.239 | 0.000 |
| CRP3 | 501 | 3.44 | 1.343 | 0.261 | 0.171 | -0.261 | 0.000 |
| CRP4 | 501 | 3.16 | 1.228 | 0.191 | 0.132 | -0.191 | 0.000 |
| CRP5 | 501 | 3.07 | 1.151 | 0.17 | 0.161 | -0.17 | 0.000 |
| CRP6 | 501 | 3.38 | 1.201 | 0.247 | 0.134 | -0.247 | 0.000 |
| PD1 | 501 | 3.01 | 1.293 | 0.209 | 0.209 | -0.194 | 0.000 |
| PD2 | 501 | 2.9 | 1.266 | 0.226 | 0.226 | -0.163 | 0.000 |
| PD3 | 501 | 2.98 | 1.397 | 0.196 | 0.196 | -0.165 | 0.000 |
| PD4 | 501 | 3.07 | 1.414 | 0.168 | 0.168 | -0.16 | 0.000 |
| PD5 | 501 | 3.08 | 1.455 | 0.166 | 0.154 | -0.166 | 0.000 |

Appendix 5: QQ plot

| Appendix 6: Cross loading result | | | | | | | |
| --- | --- | --- | --- | --- | --- | --- | --- |
|  | **AN** | **AT** | **CRP** | **GU** | **NO** | **PD** | **PEB** |
| AN1 | **0.917** | 0.436 | 0.593 | 0.481 | 0.435 | -0.009 | 0.476 |
| AN2 | **0.899** | 0.396 | 0.526 | 0.422 | 0.393 | -0.059 | 0.481 |
| AN3 | **0.894** | 0.388 | 0.515 | 0.477 | 0.444 | -0.064 | 0.495 |
| AT1 | 0.394 | **0.886** | 0.481 | 0.486 | 0.605 | 0.270 | 0.429 |
| AT2 | 0.423 | **0.880** | 0.473 | 0.467 | 0.613 | 0.273 | 0.465 |
| AT3 | 0.362 | **0.860** | 0.387 | 0.406 | 0.521 | 0.270 | 0.363 |
| CRP1 | 0.587 | 0.476 | **0.884** | 0.643 | 0.573 | 0.026 | 0.526 |
| CRP2 | 0.543 | 0.479 | **0.868** | 0.585 | 0.562 | -0.017 | 0.534 |
| CRP3 | 0.590 | 0.459 | **0.885** | 0.575 | 0.509 | 0.015 | 0.529 |
| CRP4 | 0.479 | 0.380 | **0.863** | 0.464 | 0.422 | 0.063 | 0.404 |
| CRP5 | 0.476 | 0.376 | **0.846** | 0.452 | 0.438 | 0.086 | 0.376 |
| CRP6 | 0.397 | 0.458 | **0.801** | 0.488 | 0.497 | 0.114 | 0.373 |
| GU1 | 0.471 | 0.491 | 0.590 | **0.892** | 0.579 | 0.111 | 0.455 |
| GU2 | 0.445 | 0.420 | 0.511 | **0.831** | 0.497 | 0.076 | 0.300 |
| GU3 | 0.474 | 0.461 | 0.574 | **0.890** | 0.557 | 0.109 | 0.396 |
| GU4 | 0.386 | 0.439 | 0.523 | **0.877** | 0.520 | 0.053 | 0.356 |
| NO1 | 0.428 | 0.610 | 0.548 | 0.578 | **0.907** | 0.205 | 0.434 |
| NO2 | 0.411 | 0.559 | 0.510 | 0.551 | **0.891** | 0.162 | 0.408 |
| NO3 | 0.430 | 0.627 | 0.533 | 0.542 | **0.906** | 0.212 | 0.421 |
| PD1 | -0.041 | 0.304 | 0.078 | 0.096 | 0.224 | **0.900** | -0.166 |
| PD2 | -0.019 | 0.300 | 0.055 | 0.101 | 0.189 | **0.863** | -0.182 |
| PD3 | -0.064 | 0.256 | 0.009 | 0.062 | 0.170 | **0.915** | -0.221 |
| PD4 | -0.070 | 0.252 | 0.022 | 0.080 | 0.183 | **0.908** | -0.218 |
| PD5 | -0.014 | 0.284 | 0.074 | 0.121 | 0.206 | **0.892** | -0.199 |
| PEB1 | 0.502 | 0.472 | 0.519 | 0.483 | 0.497 | -0.125 | **0.907** |
| PEB2 | 0.493 | 0.466 | 0.492 | 0.378 | 0.426 | -0.173 | **0.878** |
| PEB3 | 0.470 | 0.438 | 0.492 | 0.390 | 0.439 | -0.153 | **0.874** |
| PEB4 | 0.423 | 0.343 | 0.407 | 0.324 | 0.336 | -0.280 | **0.835** |
| PEB5 | 0.427 | 0.361 | 0.432 | 0.298 | 0.316 | -0.245 | **0.842** |

Note: The highest factor loading value of every indicator has been highlighted

| Appendix 7 Research items | | |
| --- | --- | --- |
| Source of Construct | Coding | Item |
| Guilt  (Ágoston et al. 2022) | GU1 | I feel guilty when I do something polluting that I had stopped doing before. |
|  | GU2 | I feel guilty for not paying enough attention to the issue of climate change. |
|  | GU3 | At times I feel some personal responsibility for the problems and unfolding impacts of climate change. |
|  | GU4 | It makes me feel uneasy that I am part of a system that is amplifying climate change. |
| Awe  (Shiota et al. 2006) | AWE1 | I often feel awe. |
|  | AWE2 | I have many opportunities to see the beauty of nature. |
|  | AWE3 | I seek out experiences that challenge my understanding of the world. |
| Attitude  (Qin and Hsu 2022) | AT1 | For me, performing pro-environmental behaviors is necessary |
|  | AT2 | For me, performing pro-environmental behaviors is beneficial |
|  | AT3 | For me, performing pro-environmental behaviors is wise |
| Pro-environmental behavior  (Stern 2000) | PEB1 | Would you like to separate newspapers, plastic and glass bottles? |
|  | PEB2 | Would you like to reduce your driving to protect the environment? |
|  | PEB3 | Would you like to recycle water to protect the environment? |
|  | PEB4 | Would you like to participate in environmental activities organized by charity organizations? |
|  | PEB5 | Would you like to make a donation to an environmental organization? |
| Nostalgia  (Wildschut et al. 2006) | NO1 | The COVID-19 makes me feel quite nostalgic |
|  | NO2 | The COVID-19 gives me nostalgic feelings |
|  | NO3 | The COVID-19 makes me feel nostalgia at the moment |
| Power distance  belief (Yoo et al. 2011) | PD1 | People in higher positions should make most decisions without consulting people in lower positions |
|  | PD2 | People in higher positions should not ask the opinions of people in lower positions too frequently |
|  | PD3 | People in higher positions should avoid social interaction with people in lower positions |
|  | PD4 | People in lower positions should not disagree with decisions by people in higher positions |
|  | PD5 | People in higher positions should not delegate important tasks to people in lower positions |
| COVID-19 risk perception  (O’Connor and Assaker 2022) | CRP1 | COVID-19 represents a long- lasting health threat to the people/planet |
|  | CRP2 | COVID-19 is likely to have a destructive economic impact on the people/planet |
|  | CRP3 | COVID-19 is likely to negatively affect my daily routine/life |
|  | CRP4 | COVID-19 is likely to negatively affect my finances |
|  | CRP5 | I am worried I might contract COVID-19 |
|  | CRP6 | I am worried people I know might die from COVID-19 |

Appendix 8 G Power software screen shot

**Reference**

Ágoston Csilla, Urbán Róbert, Nagy Bence, Csaba Benedek, Kőváry Zoltán, Kovács Kristóf, Varga Attila, Dúll Andrea, Mónus Ferenc, Shaw Carrie A., Demetrovics Zsolt. The psychological consequences of the ecological crisis: Three new questionnaires to assess eco-anxiety, eco-guilt, and ecological grief. Climate Risk Management 2022; 37: 100441.

O’Connor Peter, Assaker Guy. COVID-19’s effects on future pro-environmental traveler behavior: an empirical examination using norm activation, economic sacrifices, and risk perception theories. Journal of Sustainable Tourism 2022; 30 (1): 89-107.

Qin Qun, Hsu Cathy H. C. Urban travelers’ pro-environmental behaviors: Composition and role of pro-environmental contextual force. Tourism Management 2022; 92: 104561.

Shiota Michelle N., Keltner Dacher, John Oliver P. Positive emotion dispositions differentially associated with Big Five personality and attachment style. The Journal of Positive Psychology 2006; 1 (2): 61-71.

Stern Paul C. New Environmental Theories: Toward a Coherent Theory of Environmentally Significant Behavior. Journal of Social Issues 2000; 56 (3): 407-424.

Wildschut Tim, Sedikides Constantine, Arndt Jamie, Routledge Clay. Nostalgia: content, triggers, functions. Journal of personality and social psychology 2006; 91 (5): 975-993.

Yoo Boonghee, Donthu Naveen, Lenartowicz Tomasz. Measuring Hofstede's Five Dimensions of Cultural Values at the Individual Level: Development and Validation of CVSCALE. Journal of International Consumer Marketing 2011; 23 (3-4): 193-210.
